# Antimicrobial Pharmacokinetic and Pharmacodynamic Considerations in Special Populations: A Call to Action

**DOI:** 10.1093/ofid/ofag093

**Published:** 2026-03-03

**Authors:** Marguerite L Monogue, James M Sanders, Nicholas J Mercuro, Crystal K Hodge, Esther Golnabi, Tyla Carettini, Christina F Yen, James B Cutrell

**Affiliations:** US Medical Affairs, bioMérieux, Salt Lake City, Utah, USA; Department of Pharmacy, University of Texas Southwestern Medical Center, Dallas, Texas, USA; Division of Infectious Diseases and Geographic Medicine, Department of Internal Medicine, University of Texas Southwestern Medical Center, Dallas, Texas, USA; Division of Infectious Diseases, Department of Pharmacy, MaineHealth Maine Medical Center, Portland, Maine, USA; Department of Pharmacy, University of Texas Southwestern Medical Center, Dallas, Texas, USA; Department of Pharmacotherapy, University of North Texas Health Science Center, Fort Worth, Texas, USA; Department of Pharmacy, University of Texas Southwestern Medical Center, Dallas, Texas, USA; Division of Infectious Diseases and Geographic Medicine, Department of Internal Medicine, University of Texas Southwestern Medical Center, Dallas, Texas, USA; Department of Pharmacy, University of Texas Southwestern Medical Center, Dallas, Texas, USA; Division of Infectious Diseases, Department of Internal Medicine, MaineHealth Maine Medical Center, Portland, Maine, USA; Department of Medicine, Tufts University School of Medicine, Boston, Massachusetts, USA; Division of Infectious Diseases and Geographic Medicine, Department of Internal Medicine, University of Texas Southwestern Medical Center, Dallas, Texas, USA

**Keywords:** antimicrobial, cystic fibrosis, obesity, pharmacodynamics, pharmacokinetics

## Abstract

Pharmacokinetic (PK) and pharmacodynamic (PD) variability in special populations can impact antimicrobial efficacy and safety. This review highlights the importance of PK/PD optimization in patients known to have altered PK, including those with obesity, cystic fibrosis, renal dysfunction, critical illness, transplantation, pregnancy, and/or significant burns. Historically, PK/PD data are underrepresented in these populations, leading to suboptimal dosing recommendations and increased risks of therapeutic failure or toxicity. Herein, we discuss key physiological alterations affecting antimicrobial PK/PD, regulatory challenges, and currently available solutions. To bridge these knowledge gaps, we advocate for broader patient inclusion in clinical trials, improved PK modeling, real-world data collection, and increased investment in precision dosing strategies. Addressing these issues has the potential to enhance patient outcomes, reduce antimicrobial resistance, and improve infectious diseases management. This review serves as a call to action for researchers, clinicians, and policymakers to prioritize PK/PD research in special patient populations.

## Teaser

This review highlights the need to optimize antimicrobial pharmacokinetics and pharmacodynamics in special populations, including those with critical illness, renal impairment, obesity, and cystic fibrosis. A call is made for inclusive clinical trial designs, enhanced therapeutic drug monitoring, precision dosing strategies, and policy-driven incentives to address current data gaps and improve clinical outcomes.

Pharmacokinetics (PK) and pharmacodynamics (PD) are key determinants of antimicrobial exposure, but they can vary widely across different patient populations. Optimization of antimicrobial dosing based on PK/PD principles can have important implications in the clinical treatment of infections. Historically, drug development programs for novel or established antimicrobials generated limited PK data and neglected important subpopulations. This narrative review highlights special populations at risk for clinically relevant alterations in PK and calls for proactive steps to generate actionable PK/PD data in these patients.

## OVERVIEW OF PK/PD PRINCIPLES

PK describes the absorption, distribution, metabolism, and elimination of an agent through the body. PK properties, such as half-life, clearance, and volume of distribution (V_d_), determine the drug's concentration in the body over time. In infectious diseases, these concentration-time profiles correlate antimicrobial exposure with efficacy and/or toxicity, also known as PD. Antimicrobial PK/PD is traditionally categorized into 1 or more of the following PK/PD indices: (1) duration of time that the free drug concentration remains above the minimum inhibitory concentration (*f*T > MIC), (2) the ratio of the maximal free drug concentration to the MIC (*f*C_max_/MIC), and (3) the ratio of the 24-hour free area under the concentration time-curve (AUC) to the MIC (*f*AUC/MIC) [1, 2]. Quantifying the magnitude of antimicrobial exposure to maximize efficacy and minimize toxicity aids in dose determination, a key step in preclinical drug development [3, 4].

The significance of PK/PD data in preclinical studies and regulatory submissions has evolved over time, in concert with our understanding of this science [4, 5]. Historically, PK studies were conducted in healthy male volunteers, which can underestimate human variability in drug exposures due to the homogeneous population [6]. It is now appreciated that differences in sex, body weight, organ impairment, genetic polymorphisms, and food/drug interactions can alter a drug's expected PK properties. Several of these variables can be evaluated using population PK models; however, more robust PK data are needed from subpopulations of interest to more accurately predict human drug exposures associated with efficacy and toxicity [7]. Both the US Food and Drug Administration (FDA) and the European Medicines Agency now emphasize the need for clinical PK studies across diseases states and age groups at various stages of clinical development to support PK/PD analyses [8, 9].

Sole reliance on in vitro, animal, or healthy volunteer PK/PD studies for dose design and execution in later phase clinical studies has resulted in the failure of drug development programs. For example, daptomycin's lung-specific PK/PD was not studied in humans prior to clinical trials assessing safety and efficacy for the treatment of acute bacterial community-acquired pneumonia. Rather, clinical utility was assumed based on animal pneumonia models and in vitro activity against common bacterial pneumonia organisms (eg, *Streptococcus pneumoniae* and *Staphylococcus aureus*). Only after clinical studies failed was it discovered that human pulmonary surfactants drastically decreased the activity of daptomycin, explaining the poor clinical outcomes [10]. Similar stories of limited PK/PD data driving flawed decision-making in clinical trials include tigecycline for bloodstream infections, ceftobiprole for hospital- or ventilator-acquired pneumonia, doripenem for ventilator-associated pneumonia, and oral eravacycline for urinary tract infections [11–14]. The availability of robust PK/PD data to inform drug dosing and development could have mitigated some of these high-profile clinical failures. Instead, patients suffered increased clinical failures, and the antimicrobials received black-box warnings, failed to achieve approval for specific indications, or never made it to the market.

Unfortunately, the story of poor investment in PK/PD studies continues. Regulatory approved doses may not be optimized against specific infections or patient populations—an issue that is neither identifiable nor correctable until clinical failures begin to emerge. Higher doses than initially recommended are now standard for several antimicrobials (Table 1) [15].

**Table 1.** Postmarketing Changes in Standard Antimicrobial Dosing and Administration to Improve Pharmacokinetic/Pharmacodynamic Target Attainment

Agent	Approved Initial Dosage/Administration Strategy	Accepted or Suggested Updates	Indication	References
Ceftaroline	600 mg q12h, 1-h infusion	600 mg q8h, 1-h infusion	MRSA with MICs 2–4 µg/mL	[15, 16]
Ceftolozane-tazobactam	1.5 g IV q8h, 1-h infusion	3 g IV q8h, 3-h infusion	Pneumonia; MDROs	[17]
Daptomycin	4–6 mg/kg q24h	8–12 mg/kg q24h	*Enterococcus faecium*	[15, 18]
Minocycline	100 mg q12h	200 mg q12h	*Acinetobacter baumannii*	[17, 19]
Tigecycline	100 mg loading dose, 50 mg q12h	200 mg loading dose, 100 mg q12h	MDROs; severe infections	[17, 20]

This table highlights examples of antimicrobials for which postmarketing modifications to dosing or administration strategies have been adopted or suggested to improve pharmacokinetic/pharmacodynamic target attainment.

Abbreviations: IV, intravenous; MDRO, multidrug-resistant organism; MIC, minimum inhibitory concentration; MRSA, methicillin-resistant *Staphylococcus aureus*; q8h, every 8 hours; q12h, every 12 hours; q24h, every 24 hours.

This raises the question: How many patients are undertreated before we get the “right dose”? Available literature identifies several patient populations at risk for altered PK, including, but not limited to, patients with obesity, renal dysfunction, critical illness, or cystic fibrosis (CF). Identifying how antimicrobial PK differs in these populations compared with the general population is essential in anticipating risks of reduced efficacy and toxicity.

### RENAL DYSFUNCTION AND HYPERFUNCTION

Renal function is a major determinant of antimicrobial PK because of its central role in drug clearance. Chronic kidney disease (CKD) affects a substantial proportion of the US population, ranging from 6% to 38% of people. Older adults and populations with comorbidities such as diabetes and hypertension have higher rates of CKD [21]. Hyperfunction of the kidney, known as augmented renal clearance (ARC), is observed in approximately 40% of patients in intensive care units (ICUs) [22]. Acute infection-related changes in renal function may arise from direct renal involvement, immune-mediated injury, or hypoperfusion during septic shock [23].

Antimicrobials with substantial renal elimination (eg, >30% unchanged drug excreted in urine) are particularly sensitive to changes in kidney function [24]. Hydrophilic agents, such as aminoglycosides and most β-lactams, are greatly impacted by renal clearance, whereas lipophilic agents are more commonly eliminated hepatically [25]. ARC increases antimicrobial elimination, resulting in reduced systemic drug exposure, whereas CKD or acute kidney injury (AKI) decreases renal elimination, leading to increased systemic exposure and potential toxicity (Table 2).

**Table 2.** Alterations in Antimicrobial Pharmacokinetic Properties Based on Patient Population

Patient Population	Notable Changes	Examples of Antimicrobials Impacted	Antimicrobial Property Impacting PK	Key PK Parameter Changes				Impact on PTA	References
				Absorption	Distribution	Metabolism	Elimination		
Renal dysfunction	AKI, CKD	Aminoglycosides, β-lactams	Hydrophilicity	…	…	…	↓	↑	[24, 26]
Critical illness	Fluid resuscitation	Aminoglycosides, β-lactams	Hydrophilicity, protein binding	…	↑	…	…	↓	[27]
	Hypoalbuminemia	Ceftriaxone, ertapenem	Protein binding	…	↑	…	↑	↓	[28, 29]
	ECMO	Amphotericin B, meropenem	Lipophilicity, protein binding, sequestration	…	↑	…	…	↓	[30, 31]
	ARC	Aminoglycosides, β-lactams	Hydrophilicity, protein binding	…	…	…	↑	↓	[32]
Obesity	Increased mass (adipose and lean tissue)	Daptomycin	Protein binding, hydrophilicity, renal elimination	…	↑ or =	…	↑ or =	↓ or =	[33–41]
Cystic fibrosis	CFTR channel modifications, gut physiology	Azithromycin, doxycycline, linezolid	Bioavailability	↓	…	…	…	↓	[42–44]
	Increased plasma volume, urine flow	Aminoglycosides, β-lactams	Hydrophilicity	…	↑	…	↑	↓	[44, 45]
HSCT/SOT	Renal graft recovery	β-lactams	Hydrophilicity	…	…	…	↓ or ↑	↓ or ↑	[46]
	Altered gastric motility, delayed absorption	Letermovir, voriconazole, posaconazole	Bioavailability	↓	…	…	…	↓	[47–50]
	Mucositis	Posaconazole	Bioavailability	↓	…	…	…	↓	[50, 51]
	Biliary complications and reduced total bilirubin	Tetracyclines, cefotaxime	Lipophilicity	↓	…	…	↓	↓	[52–54]
	Drug–drug interactions, polymorphisms	Voriconazole	CYP450	…	…	↓ or ↑	…	↓ or ↑	[55–57]
Pregnancy	Increased stomach pH, delayed gastric emptying	Ampicillin, artesunate	Acid labile, bioavailability	↓	…	…	…	↓	[58–62]
	Volume and fluid expansion	Amoxicillin, imipenem, trimethoprim	Hydrophilicity	…	↑	…	…	↓	[61, 63–66]
	ARC	β-lactams	Hydrophilicity, time-dependent PD	…	…	…	↑	↓	[59, 63, 65]
Burn injury	Disrupted skin integrity	Aminoglycosides	Topical administration	↑	…	…	…	↑	[67–69]
	Fluid resuscitation	β-lactams, daptomycin, aminoglycosides	Hydrophilicity	…	↑	…	…	↓	[70, 71]
	Hypercatabolic state (hepatic and renal)	Piperacillin-tazobactam, ceftazidime, cefepime	CYP450, protein transporters, time-dependent PD	…	…	↑	↑	↓	[71–74]

This table summarizes key physiologic and pathophysiologic factors across special populations that contribute to clinically relevant alterations in antimicrobial pharmacokinetics.

Abbreviations: AKI, acute kidney injury; ARC, augmented renal clearance; CFTR, cystic fibrosis transmembrane conductance regulator; CKD, chronic kidney disease; ECMO, extracorporeal membrane oxygenation; HSCT, hematopoietic stem cell transplant; PD, pharmacodynamic; PK, pharmacokinetic; PTA, probability of target attainment; SOT, solid organ transplant.

Failure to achieve appropriate antimicrobial exposure in the setting of altered renal function has been associated with delayed bacterial clearance, treatment failure, and selection of resistant subpopulations [75–77]. Dose reductions in the setting of CKD and AKI are routine practice but are often informed by imprecise estimates of clearance. Dose increases or prolonged infusions in the setting of ARC are not standardized since ARC has only recently gained broader recognition [78]. Therapeutic drug monitoring (TDM) can improve dosing and exposures in patients with ARC, but access to routine TDM and the necessary infectious diseases expertise to interpret and act upon TDM data beyond vancomycin and aminoglycosides are limited [30, 79].

Conversely, upfront investment in a population PK model with ARC helped justify higher cefiderocol dosing and improved PK/PD target attainment [80]. This example highlights the need for additional research in populations with altered renal function. Accurate estimation of renal function remains a major challenge in antimicrobial dosing. There are multiple ways to calculate creatinine clearance (CrCl), such as the Modification of Diet in Renal Disease (MDRD) and Cockcroft-Gault formulas, which serve as surrogates for actual renal filtration but lead to inconsistencies in dosing recommendations [81]. Crude estimates of CrCl in patients with AKI and renal replacement therapies (RRTs) often dictate recommended antimicrobial doses [82]. In the setting of acute changes, a patient's serum creatinine may be significantly delayed in reflecting the degree of injury. Delayed recognition of renal injury may lead to drug accumulation and toxicity, whereas premature dose reduction risks inadequate exposure during the critical early phase of infection [83, 84]. Given the frequency and dynamic nature of renal dysfunction in hospitalized patients, renal-specific PK/PD considerations are essential for optimizing antimicrobial dosing and avoiding preventable treatment failure or toxicity.

### CRITICAL ILLNESS

The complex pathophysiology of critical illness alters drug PK/PD parameters [85]. Drug distribution can change during critical illness due to changes in V_d_ and hypoalbuminemia. Increases in V_d_ result from endothelial dysfunction, capillary leak, altered protein binding, and aggressive fluid resuscitation. These changes disproportionately affect hydrophilic antimicrobials, such as β-lactams, which primarily distribute within the intravascular space and may require loading doses or alternative infusion strategies to achieve adequate early exposure [27, 28].

Renal dysfunction and utilization of RRTs are common in ICUs. To address impaired kidney function and volume status, RRT is prescribed with varying dialysate and filtrate flow rates [86]. Application of continuous RRT (CRRT) utilizes different modalities: convection (continuous veno-venous hemofiltration [CVVH]), diffusion (continuous veno-venous hemodialysis [CVVHD]), or both (continuous veno-venous hemodiafiltration [CVVHDF]) [87]. The impact on antimicrobial PK depends on the modality and dose of CRRT used, but in general, utilization of CRRT increases V_d_ and clearance of antimicrobials [88]. All modalities using convection more effectively eliminate antimicrobials with high molecular weights, whereas the combination of convection and diffusion (CVVHDF) results in augmented clearance of antimicrobials compared to convection or diffusion alone [88]. Additionally, the higher the CRRT dose or effluent flow rate, the greater the drug clearance. Other drug-specific characteristics, such as lipophilicity and ionization, impact clearance as well [86]. As CRRT only eliminates the free fraction of the antimicrobials, highly protein-bound antimicrobials are less impacted by CRRT (Table 2) [89]. Notably, cefiderocol represents the first antimicrobial with dose-optimized and clinically validated CRRT dosing recommendations, supported by population PK modeling and clinical data, highlighting the feasibility and value of proactive PK/PD evaluation in patients receiving CRRT [90, 91].

Critically ill patients with cardiorespiratory failure may require extracorporeal membrane oxygenation (ECMO) for adequate blood perfusion and organ oxygenation. Due to the large surface area of ECMO circuits, lipophilic and/or highly protein-bound antimicrobials bind the circuit components, resulting in subtherapeutic plasma levels [88]. The degree of sequestration depends on the type of tubing, pump, oxygenator, and priming solution. Furthermore, the addition of priming solution can increase the V_d_ of drugs [88]. Evaluation of antimicrobials under ECMO conditions (eg, cefepime, piperacillin-tazobactam, cefiderocol) has demonstrated variable target attainment with conventional and high-dose regimens, highlighting the limitations of standard dosing in this setting [92–94].

Given these complexities, many antimicrobials are ineffectively dosed in critical illness due to variable patient PK, intra- and interinstitutional variability in antimicrobial dosing, and extracorporeal therapies used [95]. Clinical outcomes in critically ill populations demonstrate that when PK/PD targets are not attained, there is an increased risk of microbiological failure, prolonged ICU length of stay, and mortality [95–97]. Key unmet needs include defining dosing recommendations that account for extracorporeal therapies, determining when TDM meaningfully improves outcomes, and clarifying the role of model-informed precision dosing tools in routine ICU practice [98].

### OBESITY

Approximately 42% of US adults meet criteria for obesity based on body mass index. Despite this prevalence, antimicrobial PK data across obesity classes (class I–III) remain ill-defined, with most studies enrolling small cohorts that limit class-specific dosing conclusions [99–101]. Given the frequency of obesity among hospitalized patients, clarifying when obesity meaningfully alters antimicrobial exposure is increasingly relevant to routine clinical care.

The first consideration is administration route and tissue distribution. Despite a theoretical increase in intestinal perfusion and additional abdominal fat, the lack of significant bioavailability differences for oral drugs accentuates the imperative for additional PK and clinical data [101]. Concerns related to intralipomatous injections in obese patients that were intended as intramuscular injections have been raised since 1982 [101, 102]. Obesity may alter antimicrobial penetration into certain tissues, although the relationship between tissue concentrations and clinical outcomes remains poorly defined. A lipid partition coefficient can approximate the drug's ability to concentrate in adipose tissue but does not necessarily correlate to serum drug concentrations [33, 101]. Other drug distribution factors impacted by obesity include more lipoproteins to competitively bind albumin and alpha-1-acid glycoproteins [101]. As a result, weight-based dosing strategies vary by antimicrobial, with total, ideal, or adjusted body weight used (Table 3) [34].

**Table 3.** Examples of Antimicrobial Dosing Recommendations in Specific Patient Populations

Patient Population	Agent	Standard Dosage/Administration Strategy	Recommended or Proposed Updates	Rationale and Considerations	References
Renal dysfunction	Daptomycin	Intermittent HD: standard dose q48h	Intermittent HD: standard dose on 48-h interdialytic days; increase dose by 50% on the 72-h interdialytic day	Due to daptomycin’s high renal excretion, elimination is substantially slower in patients with renal dysfunction, allowing for prolonged doses between HD sessions.	[103]
Critical illness	Meropenem	1–2 g q8h, 30-min infusion	2 g q8h, 3-h infusion	Pathophysiological changes in critically ill patients can alter volume of distribution and total drug clearance, resulting in altered meropenem pharmacokinetics and variable plasma concentrations.	[104, 105]
Obesity	Acyclovir	Dose based on ABW	Dose based on adjBW	Use of ABW in obese patients resulted in adverse drug events (eg, renal failure) but use of IBW has led to inadequate exposures; the V_d_ (L/kg) does not increase in direct proportions to the increase in adipose tissue in obese patients. Limited data available.	[35, 106–108]
	Cefazolin	2 g for surgical prophylaxis	3 g of cefazolin for surgical prophylaxis in persons >120 kg	Inadequate subcutaneous tissue concentrations and a “favorable” side effect profile. A systematic review suggested that outcomes data do not warrant the use of 3 g.	[109–112]
	Linezolid	600 mg BID	600 mg TID or 900 mg BID	In obesity, the typical dose of 600 mg BID of the moderately lipophilic linezolid is insufficient to reach the target time above MIC for MRSA. The clinical relevance is debatable with few high-quality studies available.	[56, 113–119]
	Voriconazole	Dosed based on ABW	Dose based on adjBW or IBW	Use of ABW is associated with significantly higher trough levels manifesting clinically as neurotoxicity. Limited data available for empiric dosing with adjBW vs IBW. TDM recommended.	[120–124]
Cystic fibrosis	Ceftaroline	600 mg q12h, 1-h infusion	600 mg q8h, 2-h infusion	Since ceftaroline half-life is shorter in CF patients, a higher dose and longer infusion will optimize *f*T > MIC exposure.	[125]
HSCT/SOT	Cefotaxime	1 g q6h infused over 20 min	4 g as continuous infusion	Based on a small study of patients undergoing orthotopic liver transplants. Both arms had adequate biliary exposures and were able to reach serum target concentrations above the MIC for at least 60% of the dosing interval. However, some patients in the intermittent arm developed undetectable levels or insufficient levels during the reperfusion phase of the transplant surgery.	[54]
Pregnancy	Darunavir/ritonavir	800 mg/100 mg daily if treatment naive	600 mg/100 mg q12h with food	Pregnant individuals experience low darunavir exposures and do not qualify for daily dosing even if treatment naive. Instead, BID dosing is recommended. The use of the higher dose (800 mg/100 mg) does not increase darunavir exposures and is not recommended by current guidelines.	[126]
	Amoxicillin	500–875 mg PO every 8–12 h	Increase frequency (eg, every 6–8 h) or dose	Increased renal clearance during pregnancy reduces drug exposure, requiring dose adjustments to maintain therapeutic concentrations.	[60]
Burn injury	Piperacillin-tazobactam	4.5 g every 6–8 h, infused over 30 min	4.5 g q6h, prolonged infusion (≥3 h) or continuous infusion	Burn patients may have augmented renal clearance, increasing drug clearance and reducing systemic exposure for highly renally eliminated ABX. Some studies suggest higher doses beyond 18 g/d, but safety and tolerability should be considered.	[70, 127, 128]
	Meropenem	500 mg, 1 g, or 2 g IV q8h, infused over 30 min	1 g IV at 0, 4, and 8 h then 1 g q8h; consider prolonged or continuous infusion	Assuming standard MIC targets (ie, not an MDRO) and infected tissue that is not an anatomically protected site (eg, CNS), then this dosing strategy provides a higher total loading dose to accommodate the larger V_d_ of a hydrophilic drug in burn victims with prolonged infusions to optimize the time over MIC PD parameter.	[70, 72, 128]
	Ciprofloxacin	400 mg IV BID or 500 mg PO BID	400 mg IV q8h or 600 mg PO q12h	As a concentration-dependent antimicrobial, larger doses may be needed to get adequate bactericidal concentrations when patients have a larger than normal V_d_. Minimal clinical data are available to support this dosing, but there are some safety data in burn victims up to 600 mg q8h.	[70, 128–131]

This table provides examples of standard antimicrobial dosing strategies and corresponding recommended or proposed adjustments in select special populations.

Abbreviations: ABW, actual body weight; ABX, antibiotic; adjBW, adjusted body weight; BID, twice daily; CF, cystic fibrosis; CNS, central nervous system; *f*T, free time; HD, hemodialysis; HSCT, hematopoietic stem cell transplant; IBW, ideal body weight; IV, intravenous; MDRO, multidrug-resistant organism; MIC, minimum inhibitory concentration; MRSA, methicillin-resistant *Staphylococcus aureus*; PD, pharmacodynamic; PO, oral; q6h, every 6 hours; q8h, every 8 hours; q12h, every 12 hours; q24h, every 24 hours; q48h, every 48 hours; SOT, solid organ transplant; TDM, therapeutic drug monitoring; TID, 3 times daily; V_d_, volume of distribution.

The impact of obesity on metabolism is complex. Despite no substantial difference in hepatic blood flow, enzymatic damage due to excessive fatty acid deposits in obesity can reduce antimicrobial metabolism [101]. Phase I metabolism is mostly unaffected by obesity; phase II may be increased in obesity [33]. The degree to which predicted renal clearance is impacted in obesity varies by the specific equation used. Lean and ideal body weight appear more accurate than total body weight in CrCl and estimated glomerular filtration rate calculations, although neither has a perfectly linear correlation into the class III obesity range. Corrective equations for obesity such as the Salazar-Corcoran and 4-variable modification of MDRD (MDRD4) also lack precision and vary drastically by obesity classification [101].

In a retrospective cohort, obese patients receiving β-lactams experienced higher rates of clinical treatment failure and longer hospitalization compared with nonobese patients [132]. Toxicity considerations are also relevant, as data have shown obesity as a risk factor for vancomycin-associated nephrotoxicity [133]. Given PK changes in obesity, TDM may help optimize exposure and improve outcomes in high-risk infections; however, serum concentrations do not always correlate with tissue exposure [113, 120]. These gaps in knowledge related to the implications of obesity on antimicrobial dosing will have increasing clinical importance given its rising prevalence in the population.

### CYSTIC FIBROSIS

People with CF (PwCF) experience more infections and consequently antimicrobial exposures than non-PwCF [134, 135]. Therefore, optimization of PK/PD in this population is essential to improve antimicrobial efficacy and safety. While information varies on PK changes in PwCF, we highlight key PK considerations; a more extensive review may be found in previous publications [136–138].

Prior studies have described PwCF PK alterations in drug absorption, V_d_, plasma protein binding, metabolism, and elimination [138]. Alterations are specific to the antimicrobial with select agents displaying similar PK to non-PwCF comparators, while other antimicrobials (eg, β-lactams and aminoglycosides) have quite distinct PKs with noted increases in V_d_ and clearance. Due to cystic fibrosis transmembrane conductance regulator (CFTR) channel modifications, diet, and antimicrobial exposure changing intestinal inflammation and gut microbiome, PwCF experience altered gut physiology that may impair the absorption of oral antimicrobials [138, 139]. Several studies have shown potential delayed time to maximum concentrations without subsequent changes in overall bioavailability (Table 2) [42, 43, 140].

Several strategies to optimize antimicrobial dosing have been explored (Table 3). The established practice of TDM for tobramycin and vancomycin may promote their safety and efficacy, but the optimal sampling approach remains poorly defined [45, 141, 142]. One recent study suggests that AUC-based vancomycin dosing may improve its safety profile in PwCF [143]. Extended and continuous infusions of β-lactams are commonly employed to promote better PK/PD target attainment, but limited evidence exists to suggest a clear benefit on clinical outcomes [44, 144]. In addition, continuous infusion approaches alone may lead to insufficient concentrations in approximately 50% of patients, lending further support for TDM [133]. A combination strategy of TDM with continuous infusion approach has been explored for ceftolozane-tazobactam [145]. More recent PK/PD studies of ceftolozane-tazobactam and ceftaroline suggest that more “aggressive” dosing strategies via higher doses (eg, 3 g for ceftolozane-tazobactam) and increased frequency of administration (eg, every 8 hours for ceftaroline) are necessary to optimize PK/PD in PwCF [125, 146].

The use of inhaled antimicrobials offers the potential to increase target pulmonary exposure and reduce systemic exposure, which could optimize target PK/PD and limit toxicity. Many factors influence the lung distribution of inhaled antimicrobials and variable exposures may occur [147–149]. Several antimicrobials are FDA approved (eg, tobramycin) and additional antimicrobials (eg, ceftazidime) are used “off-label” to manage pulmonary exacerbations [150]. However, further studies are needed to ensure optimal delivery methods, dosing efficacy, and safety in prevention and treatment strategies in PwCF [150].

Historically, PwCF are underrepresented or excluded from registrational trials, limiting our knowledge surrounding novel therapeutics. Fortunately, more recent trials have provided an understanding of novel antimicrobials’ PK in this population; however, several research gaps related to PK/PD remain [151, 152]. Notably, future novel antimicrobials with a spectrum inclusive of multidrug-resistant organisms (MDROs) commonly seen in CF should be addressed during the traditional approval process. Other key areas of focus include future PK studies of approved antimicrobials (eg, cefiderocol, meropenem-vaborbactam, and lefamulin), PK/PD and efficacy studies of oral and inhaled therapies, the role of TDM in PwCF, the relationship between CFTR modulators and antimicrobials (eg, drug–drug interactions and infection prevention/treatment), and the interplay between combination therapy and its effects on PK/PD optimization.

### OTHER NOTABLE POPULATIONS

Other important populations with significant PK alterations include transplant candidates or recipients, pregnant persons, and patients with significant burns. Key high-level PK changes in each of these populations are reviewed below.

PK variations in solid organ transplant candidates and recipients are related to pathophysiologic changes from end-organ damage, graft function, immunosuppression, and several other factors. Chemotherapy and radiation therapy in hematopoietic stem cell transplant recipients introduce additional temporary PK alterations [153, 154]. Posttransplant PK changes are most consequential in the first month posttransplant, including decreased bioavailability and delayed absorption of oral antimicrobials [47, 155]. Transplant recipients also frequently encounter drug–drug interactions and medications susceptible to metabolism and elimination PK changes. Polymorphisms of metabolic alleles, particularly CYP3A5 and CYP2C19, have demonstrated significant PK alterations in transplant recipients [55, 156–158]. Additionally, the type of graft transplanted can impact the recipient's PK. For example, renal transplant recipients will have fluctuating antimicrobial concentrations as graft function recovery influences renal drug elimination [159–162]. Surgical complications, immune-mediated injury, and nephrotoxic concomitant therapies further contribute to highly variable renal clearance, complicating empiric antimicrobial dosing in this population [156, 159, 161].

Some of the more dramatic changes in PK occur during pregnancy and differ depending on trimester [163]. Absorption can be altered by the increased stomach pH and delayed gastric emptying that occur in pregnancy [58, 59, 63]. Antimicrobial distribution in pregnant women is also variable. For example, hypoalbuminemia is a common occurrence in pregnancy that changes the free drug availability of highly protein-bound medications [58, 59, 63]. Concentration-dependent antimicrobials are also diluted in pregnancy given the expansion of plasma volume and additional amniotic fluid. As the pregnancy progresses and during the postpartum period, fat tissue increases with differential tissue distributions, potentially altering lipophilic antimicrobial distribution to infected tissues. Increased antimicrobial elimination can occur through breastfeeding, with significantly increased blood flow to the mammary arteries [59, 63]. Furthermore, antimicrobial dosing in the setting of ARC, discussed in general above, represents an important consideration specifically in pregnancy [58, 163]. Pregnant women develop ARC to a CrCl of 150 mL/minute or more in the first trimester [58, 59, 63]. Conversely, hepatic metabolism and elimination in pregnancy are variable [59, 63]. The FDA has made substantial updates to pregnancy, lactation, and fertility drug labeling. However, these updates are mostly limited to animal data, limiting the ability to make exposure-based dosing recommendations [164].

Clinically relevant PK changes also occur when burns exceed 20% or more of the total body surface area [70]. In burn patients, oral absorption can be impaired by significant peripheral and gastric blood flow constriction while disrupted skin integrity alters topical absorption [70]. Burn-induced cytokine responses may trigger life-threatening hemodynamic shifts, increased capillary permeability, and myocardial depression contributing to extensive V_d_ changes [70, 71]. Burn management also requires aggressive fluid resuscitation, further diluting antimicrobials. Additional shifts of protein-rich fluids to the interstitial space increases the amount of free drug for highly protein-bound antimicrobials (eg, ceftriaxone) [70, 71]. Patients with burns more reliably exhibit a hypercatabolic state starting about 5 days post–thermal injury. Elimination is also variable with renal damage occurring in roughly a third of burn patients, with the remaining patients prone to ARC [70, 71].

Scrutiny of the PK heterogeneity in these special populations is essential to defining the therapeutic window of antimicrobials. There are insufficient PK studies in these select populations, significantly limiting available information to guide tailored antimicrobial dosing and forcing reliance on broader population PK and therapeutic indices. Inconsistent PK and drug interactions alone support the need for antimicrobial TDM and further research into its application.

### CALL TO ACTION

In most historical clinical trials, standard antimicrobial doses and infusion rates were applied across all populations with little consideration of patient- or organism-specific factors [7]. As the special populations discussed above expand, there exists heightened concern that relying on traditional antimicrobial dosing alone will lead to suboptimal patient outcomes [165]. In the following sections, we propose several action items to address this challenge.

### Encourage Broader Inclusion Criteria in Antimicrobial Registration Trials to Inform Optimal Dosing in Special Populations

**Figure 1.**
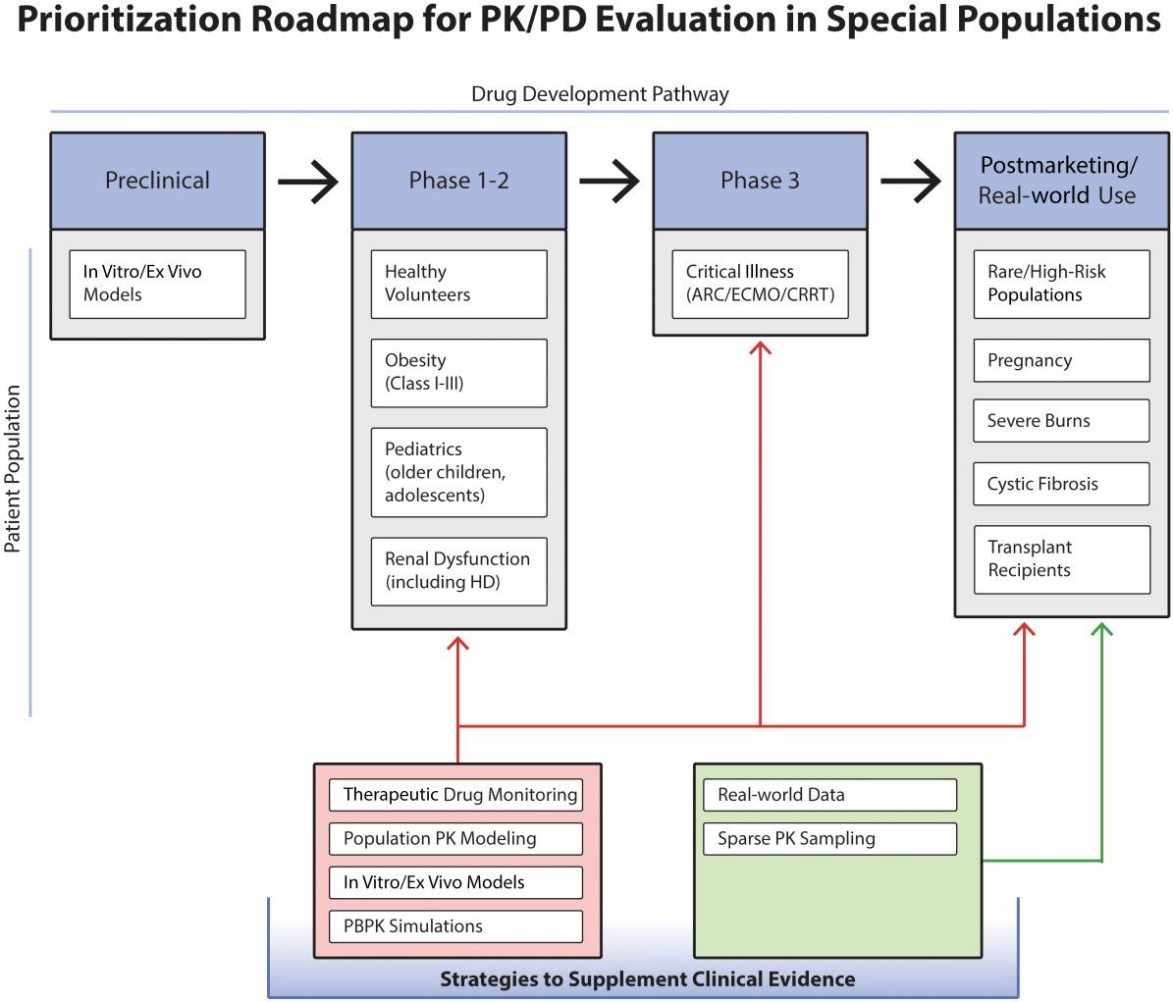
Prioritization roadmap for PK/PD evaluation in special populations during antimicrobial development. A framework for incorporating special population PK/PD data across the antimicrobial development lifecycle is shown. Early development focuses on populations with predictable physiologic alterations and high feasibility, while later phases expand to more complex or vulnerable populations. A combination of modeling, therapeutic drug monitoring, and real-world data approaches enables scalable and ethical data generation to inform dosing and labeling decisions. Abbreviations: ARC, augmented renal clearance; CRRT, continuous renal replacement therapy; ECMO, extracorporeal membrane oxygenation; HD, hemodialysis; PBPK, physiologically based pharmacokinetic (modeling); PK, pharmacokinetic.

While pharmacotherapy has made significant progress in characterizing optimal antimicrobial dosages and exposures, many of the applied recommendations in these groups are derived from small studies or extrapolated. As illustrated in Figure 1, early and intentional inclusion of select special populations within antimicrobial development programs represents a high-feasibility opportunity to generate actionable PK data before registrational trials are complete. Newer compounds, such as cefiderocol, meropenem-vaborbactam, and aztreonam-avibactam, have been studied as a prolonged infusion coupled with PK sampling, which provided additional data for dose optimization [166, 167]. Another complementary approach would be broader inclusion criteria for enrollment in clinical trials, particularly in special populations (eg, CF, ECMO) to gather actionable PK data [168]. Although direct links between PK/PD optimization and clinical outcomes remain limited for many special populations, established exposure–response relationships have been sufficient to inform dosing recommendations and regulatory labeling in select settings. However, when exposure–response relationships are uncertain or dosing strategies substantially alter clinical practice, prospective clinical outcomes data may be necessary to support broader adoption and guideline endorsement [169].

#### Create Market Incentives for Drug Development to Generate PK and Outcomes Data in Special Populations

The financial costs and barriers of bringing a new antimicrobial through research and development are significant, and anti-infectives are not considered a highly profitable therapeutic class. In response to this, the US Congress enacted the Generating Antibiotics Incentives Now (GAIN) Act, which provides additional benefits to pharmaceutical companies for developing novel products that serve a critical need in treating life-threatening and MDROs. Antimicrobials can be placed under application for qualified infectious diseases product status, which grants an additional 5 years of market exclusivity as a financial incentive [170]. However, most antimicrobials enter the market based on trials in less serious, uncomplicated infections, rather than for the indications needed in actual clinical practice [171, 172]. One recent positive development is randomized trials of novel β-lactam/β-lactamase inhibitors and other compounds to generate best-practice guidance for treatment of infections caused by MDROs [166, 167]. The additional time and expense of studying the PK and outcomes in special populations carries similar challenges. Given the high morbidity and mortality these populations face, they deserve the same level of urgency for generating high-quality PK and outcomes data. Like the MDRO space, researchers will face significant enrollment challenges, and there are no market-entry incentives to evaluate infected PwCF, patients with ARC, or other special groups in antimicrobial development. However, the FDA does recommend separate PK studies in patients with renal dysfunction if it is suspected to be significantly altered. A major priority in the National Action Plan for Combating Antimicrobial Resistance (CARB) includes the advancement of in vitro, animal model, and PK studies to facilitate antimicrobial drug development [173, 174]. Furthermore, the Society of Infectious Diseases Pharmacists has also emphasized the importance of economic incentives to support antimicrobial drug development, calling for policy-driven strategies to ensure continued innovation in this field [175].

#### Organize Private–Academic Partnerships and Research Networks to Facilitate Patient Recruitment for Special Populations in Postmarketing PK Studies

To answer unmet needs in PK/PD and outcomes data, clinicians have created networks to quickly recruit and study patients in special populations [176]. Collaboratives from private–academic partnerships, corporations, and organizational research networks have generated important PK/PD safety and efficacy data that inform dosing [7, 177]. Limited funding, time, and resources along with recruitment challenges and clinical confounders are among the most difficult obstacles researchers face when studying rare patient groups at high risk for morbidity and mortality. However, support is available through government, nonprofit organizations, and industry to conduct PK and outcomes studies in these groups [178]. The use of real-world, pragmatic randomized designs to answer challenging clinical questions has also demonstrated critical value in their ability to rapidly scale high-quality data in large populations [179].

#### Utilize In Vitro and In Silico Modeling to Simulate PK Parameters in Special Populations or in Artificial Organ Support Settings

Another potential solution to the lack of readily available patients with rare conditions that can be efficiently sampled for PK analyses is to turn to in vitro and in silico modeling to simulate conditions at target sites or in the setting of organ support therapies [91]. Improvements in these models have substantially increased understanding of PK/PD interactions, without the risks associated with in vivo PK sampling [180–182]. However, in vitro effects do not necessarily predict in vivo efficacy [166, 183]. Furthermore, there are challenges in replicating and interpreting these models for human infections including, but not limited to, interpatient variability in clearance/distribution, presence of blood and blood proteins, and longer dosing periods.

#### Expand Access and Efficiency of TDM in Special Populations to Promote Bedside PK/PD Applications

Broader application of TDM should be pursued in patients with unpredictable PK, patients with serious infections, and patients receiving antimicrobials with actionable and narrow therapeutic and safety indexes. These groups certainly include patients with altered V_d_ and clearance such as those with CF, ARC, burns, and/or support from extracorporeal modalities [184]. Triazole antifungals, aminoglycosides, and glycopeptides have paved the way by demonstrating how defined safety and efficacy targets can improve patient care [185]. Clinical data continue to emerge for TDM in other classes such as β-lactams, fluoroquinolones, echinocandins, and linezolid [177, 184, 186, 187]. Unfortunately, most novel antimicrobials require high-performance liquid chromatography and mass spectrometry for TDM, which few US centers, let alone low- and middle-income countries, have direct access to on site. Advent of broader antibiotic assays with faster turnaround time and lower costs would facilitate precision dosing in special populations across diverse global healthcare settings [188].

#### Integrate Special Population Dosing Into Clinical Guidelines

Clinical practice guidelines are a readily available resource to many providers. While select guidelines acknowledge dosing considerations for certain special populations, PK/PD guidance across infections and patient populations remains limited [189–191]. Incorporation of antimicrobial dosing recommendations for special populations, including guidance on dose adjustment, infusion strategies, and use of TDM, is needed to drive adoption at the institutional level. Several high-quality reviews and consensus documents provide a framework for special population dosing, though their integration into routine practice and clinical guidelines remains inconsistent (Table 4).

**Table 4.** Key Resources or Reviews

Patient Population	Resources or Reviews [Reference]	Notable Content
Renal dysfunction	Antibiotic dosing for critically ill adult patients receiving intermittent hemodialysis, prolonged intermittent renal replacement therapy, and continuous renal replacement therapy: an update (2020) [192]	PK considerations specific to the renal replacement modality ABX dosing recommendations in critically ill patients receiving intermittent hemodialysis, prolonged intermittent renal replacement therapy, and CRRT
	An ex vivo model to determine transmembrane clearance of antimicrobials during continuous renal replacement therapy (2025) [193] Clinical validation of antimicrobial dosing regimens for continuous renal replacement therapy based on an ex vivo dosing algorithm (2025) [182]	An ex vivo CRRT model of cefepime, meropenem, levofloxacin, and micafungin to determine the adsorption and transmembrane clearance across various hemofilters, modes, and effluent flow rates These models were clinically validated and can be used to develop appropriate dosing regimens
Critically ill	Optimization of the treatment with beta-lactam antibiotics in critically ill patients—guidelines from the French Society of Pharmacology and Therapeutics (2019) [194]	French guideline with recommendations on renal function calculations, plasma protein measurements, β-lactam PK/PD targets, and administration techniques
	Pharmacokinetics of commonly used antimicrobials in critically ill adults during extracorporeal membrane oxygenation: a systematic review (2021) [195]	Summary of available literature and dosing recommendations for ABX in ECMO patients (vancomycin, β-lactams, amikacin, linezolid, caspofungin)
	Pharmacokinetics, pharmacodynamics, and dosing considerations of novel β-lactams and β-lactam/β-lactamase inhibitors in critically ill adult patients: focus on obesity, augmented renal clearance, renal replacement therapies, and extracorporeal membrane oxygenation (2022) [94]	PK/PD exposures of ceftolozane-tazobactam, ceftazidime-avibactam, cefiderocol, ceftobiprole, imipenem-relebactam, and meropenem-vaborbactam
	Antibiotic dose optimisation in the critically ill: targets, evidence and future strategies (2024) [196]	Challenges related to inadequate antibiotic dosing in critically ill patients Summary of limitations of currently available data
	A narrative review on antimicrobial dosing in adult critically ill patients on extracorporeal membrane oxygenation (2024) [197]	Comprehensive review of the PK/PD impact ECMO poses on various antimicrobials
Obesity	Antimicrobial dosing in obese patients (1997) [33]	Historical review of medications with narrow therapeutic windows from before the obesity epidemic
	Demystifying drug dosing in obese patients (2015) [101]	Pathophysiologic changes in obesity in general Drug class chapters with literature review and drug-specific examples of clinical implementation
	Comprehensive guidance for antibiotic dosing in obese adults (2017) [118]	Obesity-specific dosing guidance
	Updated antimicrobial dosing recommendations for obese patients (2024) [34]	Comprehensive review of PK in obesity Obesity-specific dosing guidance
	Sanford Guide (Yearly) [198]	Obesity dose adjustments table with recommended dosing and citations Table includes drugs that do not need to be adjusted Updated regularly
	The pharmacokinetics of antibiotics in patients with obesity: a systematic review and consensus guidelines for dose adjustments (2025) [199]	Systematic review that found modest β-lactam PK changes in obesity without evidence to support routine dose adjustment Aminoglycosides and glycopeptides demonstrate clinically relevant PK alterations warranting weight-based dosing Evidence quality across antibiotic classes was low to very low Therapeutic drug monitoring is recommended as a pragmatic approach when obesity-specific PK data are limited
Cystic fibrosis	The pharmacokinetics of antibiotics in cystic fibrosis (2021) [138]	Discussion on how CF-related physiological changes can alter the PK of various antibiotics
	Population pharmacokinetic modeling of cefepime, meropenem, and piperacillin-tazobactam in patients with cystic fibrosis (2025) [200]	PTA of common β-lactam antimicrobials in PwCF Table with suggested dose and infusion time based on age
HSCT/Transplant	Clinical pharmacokinetics in organ transplant patients (1989) [53]	PK overview and differences by organ
	Pharmacokinetics of drugs in adult living donor liver transplant patients: regulatory factors and observations based on studies in animals and humans (2016) [52]	PK-based approach to alterations in liver transplant Summary tables of PK changes Nonantimicrobial drug-specific review
	Peri- and postsurgical evaluations of renal transplant (2017) [159]	Review of posttransplant complications and physiologic alterations that ultimately impact PK
Pregnancy	Physiologic and pharmacokinetic changes in pregnancy (2014) [62]	Systems-based review of physiologic changes in pregnancy and their PK implications
	Pregnancy-associated changes in pharmacokinetics: a systematic review (2016) [63]	Systematic review with rigorous quality assessment Summary tables with drug-specific examples and citations
	Gestation-specific changes in the anatomy and physiology of healthy pregnant women: an extended repository of model parameters for physiologically based pharmacokinetic modeling in pregnancy (2017) [59]	Review of physiologic changes in pregnancy-specific PK equation modifications based on the literature
	Drugs in pregnancy: pharmacologic and physiologic changes that affect clinical care (2020) [201]	PK approach to reviewing PK changes Enzyme-specific summaries for metabolic changes in pregnancy
	The pharmacokinetics and target attainment of antimicrobial drugs throughout pregnancy: parts I and III (2023) [61, 66]	Drug class–based review of the literature Dedicated paper to penicillins with illustration summary of PK changes Part III has Table 10, which reviews pregnancy risk in animal and human studies
	A review of antibiotic safety in pregnancy—2025 update (2025) [202]	Review of antibiotic safety data in pregnancy. Focus of the review is on newly approved antibiotics since prior 2015 publication
Burn injury	Influence of burns on pharmacokinetics and pharmacodynamics of drugs used in the care of burn patients (2008) [203]	Overview of physiologic changes Drug class–based review
	Intravenous antibiotic and antifungal agent pharmacokinetic-pharmacodynamic dosing in adults with severe burn injury (2016) [128]	PK changes and drug review Drug dosing suggestions based on PTA for specific MICs
	The biochemical alterations underlying post-burn hypermetabolism (2017) [204]	Review of biochemical mechanisms driving PK alterations
	The effects of major burn related pathophysiological changes on the pharmacokinetics and pharmacodynamics of drug use: an appraisal utilizing antibiotics (2018) [70]	Overview of physiologic changes with illustration for PK implications PK-based review of changes Alternative dosing strategies
	Pharmacokinetics and pharmacodynamics of antimicrobial agents in burn patients (2021) [71]	PK changes overview Drug class–based review

This table compiles key reviews, guidelines, and foundational resources that inform antimicrobial dosing in special populations.

Abbreviations: ABX, antibiotic; CF, cystic fibrosis; CRRT, continuous renal replacement therapy; ECMO, extracorporeal membrane oxygenation; MIC, minimum inhibitory concentration; PD, pharmacodynamic; PK, pharmacokinetic; PTA, probability of target attainment; PwCF, people with cystic fibrosis.

## CONCLUSIONS

A significant and increasing proportion of patients with serious infections fall into a population at risk for altered PK parameters. These special patient populations—including those with critical illness, CF, altered renal function, obesity, transplantation, burns, or pregnancy—are underrepresented in the available literature and clinical trials data that inform our current antimicrobial dosing. This lack of actionable clinical and PK/PD data is a major obstacle to optimization of antimicrobial therapy for these patients. To address this clinically important gap in knowledge, we call on regulatory agencies, the pharmaceutical industry, and the academic community to partner on a multipronged approach to remedy these research gaps to improve the care of these important populations.

## References

[1] Craig WA . Pharmacokinetic/pharmacodynamic parameters: rationale for antibacterial dosing of mice and men. Clin Infect Dis 1998; 26:1–10; quiz 11–2. 9455502 10.1086/516284

[2] Ambrose PG, Bhavnani SM, Rubino CM, et al Pharmacokinetics–pharmacodynamics of antimicrobial therapy: it's not just for mice anymore. Clin Infect Dis 2007; 44:79–86. 17143821 10.1086/510079

[3] Jorda A, Zeitlinger M. Preclinical pharmacokinetic/pharmacodynamic studies and clinical trials in the drug development process of EMA-approved antibacterial agents: a review. Clin Pharmacokinet 2020; 59:1071–84. 32356105 10.1007/s40262-020-00892-0 PMC7467913

[4] Palmer ME, Andrews LJ, Abbey TC, et al The importance of pharmacokinetics and pharmacodynamics in antimicrobial drug development and their influence on the success of agents developed to combat resistant gram negative pathogens: a review. Front Pharmacol 2022; 13:888079. 35959440 10.3389/fphar.2022.888079 PMC9359604

[5] Tuntland T, Ethell B, Kosaka T, et al Implementation of pharmacokinetic and pharmacodynamic strategies in early research phases of drug discovery and development at Novartis Institute of Biomedical Research. Front Pharmacol 2014; 5:174. 25120485 10.3389/fphar.2014.00174 PMC4112793

[6] Karakunnel JJ, Bui N, Palaniappan L, et al Reviewing the role of healthy volunteer studies in drug development. J Transl Med 2018; 16:336. 30509294 10.1186/s12967-018-1710-5 PMC6278009

[7] Alshaer MH, Goutelle S, Santevecchi BA, et al Cefepime precision dosing tool: from standard to precise dose using nonparametric population pharmacokinetics. Antimicrob Agents Chemother 2022; 66:e0204621. 34902271 10.1128/aac.02046-21 PMC8846452

[8] European Medicines Agency . Guideline on the use of pharmacokinetics and pharmacodynamics in the development of antimicrobial medicinal products. London: European Medicines Agency, 2016.

[9] Center for Drug Evaluation and Research . Division of Pharmacometrics. Silver Spring, MD: US Food and Drug Administration, 2021.

[10] Silverman JA, Mortin LI, VanPraagh AD, Li T, Alder J. Inhibition of daptomycin by pulmonary surfactant: in vitro modeling and clinical impact. J Infect Dis 2005; 191:2149–52. 15898002 10.1086/430352

[11] Prasad P, Sun J, Danner RL, et al Excess deaths associated with tigecycline after approval based on noninferiority trials. Clin Infect Dis 2012; 54:1699–709. 22467668 10.1093/cid/cis270 PMC3404716

[12] Lagacé-Wiens PRS, Rubinstein E. Pharmacokinetic and pharmacodynamics evaluation of ceftobiprole medocaril for the treatment of hospital-acquired pneumonia. Expert Opin Drug Metab Toxicol 2013; 9:789–99. 23590397 10.1517/17425255.2013.788150

[13] Kollef MH, Réa-Neto Á, Wunderink RG, et al Doripenem for treating nosocomial pneumonia and ventilator-associated pneumonia—authors' reply. Lancet Infect Dis 2020; 20:20–1. 10.1016/S1473-3099(19)30717-0 31876489

[14] Tetraphase Pharmaceuticals, Inc. Efficacy and Safety Study of Eravacycline Compared With Levofloxacin in Complicated Urinary Tract Infections (cUTI) . ClinicalTrials.gov. Identifier: NCT01978938. https://clinicaltrials.gov/study/NCT01978938. Accessed 4 March 2026.

[15] Clinical and Laboratory Standards Institute (CLSI) . EM100 connect—CLSI M100 ED33. Wayne, PA: CLSI, **2023**.

[16] Burnett YJ, Echevarria K, Traugott KA. Ceftaroline as salvage monotherapy for persistent MRSA bacteremia. Ann Pharmacother 2016; 50:1051–9. 27520326 10.1177/1060028016664361

[17] Tamma PD, Heil EL, Justo JA, et al Infectious Diseases Society of America 2024 guidance on the treatment of antimicrobial-resistant gram-negative infections [manuscript published online ahead of print 7 August 2024]. Clin Infect Dis 2024. doi:10.1093/cid/ciae403 39108079

[18] Turnidge J, Kahlmeter G, Cantón R, et al Daptomycin in the treatment of enterococcal bloodstream infections and endocarditis: a EUCAST position paper. Clin Microbiol Infect 2020; 26:1039–43. 32353412 10.1016/j.cmi.2020.04.027

[19] Zhou J, Ledesma KR, Chang K-T, et al Pharmacokinetics and pharmacodynamics of minocycline against *Acinetobacter baumannii* in a neutropenic murine pneumonia model. Antimicrob Agents Chemother 2017; 61:e02371-16. 28264853 10.1128/AAC.02371-16 PMC5404596

[20] Zha L, Pan L, Guo J, et al Effectiveness and safety of high dose tigecycline for the treatment of severe infections: a systematic review and meta-analysis. Adv Ther 2020; 37:1049–64. 32006240 10.1007/s12325-020-01235-y PMC7223407

[21] Centers for Disease Control and Prevention. Chronic kidney disease: common, serious, and costly. U.S. Department of Health and Human Services. https://www.cdc.gov/kidneydisease/publications-resources/ckd-national-facts.html. Accessed 4 March 2026.

[22] Hefny F, Stuart A, Kung JY, et al Prevalence and risk factors of augmented renal clearance: a systematic review and meta-analysis. Pharmaceutics 2022; 14:445. 35214177 10.3390/pharmaceutics14020445 PMC8878755

[23] Herberg J, Pahari A, Walters S, Levin M. Infectious diseases and the kidney. In: Avner ED, Harmon WE, Niaudet P, Yoshikawa N, eds. *Pediatric Nephrology*. Springer; 2009:1235–73. doi:10.1007/978-3-540-76341-3_5219153776

[24] Monogue M . Pharmacokinetic/pharmacodynamic considerations for urinary tract infections. ACCP Infectious Diseases Self-Assessment Program (IDSAP). Lenexa, KS: American College of Clinical Pharmacy;2022.

[25] Shah S, Barton G, Fischer A. Pharmacokinetic considerations and dosing strategies of antibiotics in the critically ill patient. J Intensive Care Soc 2015; 16:147–53. 28979397 10.1177/1751143714564816 PMC5606477

[26] Saran S, Rao NS, Azim A. Drug dosing in critically ill patients with acute kidney injury and on renal replacement therapy. Indian J Crit Care Med 2020; 24:129–34. 10.5005/jp-journals-10071-23392 PMC7347056 32704220

[27] Charlton M, Thompson JP. Pharmacokinetics in sepsis. BJA Educ 2019; 19:7–13. 33456848 10.1016/j.bjae.2018.09.006 PMC7807908

[28] Woodcock TE, Woodcock TM. Revised Starling equation and the glycocalyx model of transvascular fluid exchange: an improved paradigm for prescribing intravenous fluid therapy. Br J Anaesth 2012; 108:384–94. 22290457 10.1093/bja/aer515

[29] Dhanani JA, Ahern B, Lupinsky L, et al Comparative plasma pharmacokinetics of ceftriaxone and ertapenem in normoalbuminemia, hypoalbuminemia, and albumin replacement in a sheep model. Antimicrob Agents Chemother 2020; 64:e02584-19. 32366707 10.1128/AAC.02584-19 PMC7318000

[30] Kriegl L, Hatzl S, Schilcher G, et al Antifungals in patients with extracorporeal membrane oxygenation: clinical implications. Open Forum Infect Dis 2024; 11:ofae270. 10.1093/ofid/ofae270 PMC11181180 38887481

[31] Kühn D, Metz C, Seiler F, et al Antibiotic therapeutic drug monitoring in intensive care patients treated with different modalities of extracorporeal membrane oxygenation (ECMO) and renal replacement therapy: a prospective, observational single-center study. Crit Care 2020; 24:664. 33239110 10.1186/s13054-020-03397-1 PMC7689974

[32] Roger C . Understanding antimicrobial pharmacokinetics in critically ill patients to optimize antimicrobial therapy: a narrative review. J Intensive Med 2024; 4:287–98. 39035618 10.1016/j.jointm.2023.12.007 PMC11258509

[33] Wurtz R, Itokazu G, Rodvold K. Antimicrobial dosing in obese patients. Clin Infect Dis 1997; 25:112–8. 9243045 10.1086/514505

[34] Castro-Balado A, Varela-Rey I, Mejuto B, et al Updated antimicrobial dosing recommendations for obese patients. Antimicrob Agents Chemother 2024; 68:e0171923. 38526051 10.1128/aac.01719-23 PMC11064535

[35] Polso AK, Lassiter JL, Nagel JL. Impact of hospital guideline for weight-based antimicrobial dosing in morbidly obese adults and comprehensive literature review. J Clin Pharm Ther 2014; 39:584–608. 25203631 10.1111/jcpt.12200

[36] Dvorchik BH, Damphousse D. The pharmacokinetics of daptomycin in moderately obese, morbidly obese, and matched nonobese subjects. J Clin Pharmacol 2005; 45:48–56. 15601805 10.1177/0091270004269562

[37] Bookstaver PB, Bland CM, Qureshi ZP, et al Safety and effectiveness of daptomycin across a hospitalized obese population: results of a multicenter investigation in the southeastern United States. Pharmacotherapy 2013; 33:1322–30. 23712701 10.1002/phar.1298

[38] Fox AN, Smith WJ, Kupiec KE, et al Daptomycin dosing in obese patients: analysis of the use of adjusted body weight versus actual body weight. Ther Adv Infect Dis 2019; 6:2049936118820230. 30728962 10.1177/2049936118820230 PMC6354309

[39] Meng L, Mui E, Ha DR, et al Comprehensive guidance for antibiotic dosing in obese adults: 2022 update. Pharmacother J Hum Pharmacol Drug Ther 2023; 43:226–46. 10.1002/phar.2769 36703246

[40] Mellon G, Hammas K, Burdet C, et al Population pharmacokinetics and dosing simulations of amoxicillin in obese adults receiving co-amoxiclav. J Antimicrob Chemother 2020; 75:3611–8. 32888018 10.1093/jac/dkaa368

[41] Smit C, van Schip AM, van Dongen EPA, et al Dose recommendations for gentamicin in the real-world obese population with varying body weight and renal (dys)function. J Antimicrob Chemother 2020; 75:3286–92. 32785707 10.1093/jac/dkaa312 PMC7566361

[42] Beringer PM, Owens H, Nguyen A, et al Pharmacokinetics of doxycycline in adults with cystic fibrosis. Antimicrob Agents Chemother 2012; 56:70–4. 22024822 10.1128/AAC.05710-11 PMC3256044

[43] Beringer P, Huynh KMT, Kriengkauykiat J, et al Absolute bioavailability and intracellular pharmacokinetics of azithromycin in patients with cystic fibrosis. Antimicrob Agents Chemother 2005; 49:5013–7. 16304166 10.1128/AAC.49.12.5013-5017.2005 PMC1315964

[44] Hong LT, Liou TG, Deka R, et al Pharmacokinetics of continuous infusion beta-lactams in the treatment of acute pulmonary exacerbations in adult patients with cystic fibrosis. Chest 2018; 154:1108–14. 29908155 10.1016/j.chest.2018.06.002 PMC6689083

[45] Hemmann B, Woods E, Makhlouf T, et al Impact of patient-specific aminoglycoside monitoring for treatment of pediatric cystic fibrosis pulmonary exacerbations. J Pediatr Pharmacol Ther 2022; 27:655–62. 36186239 10.5863/1551-6776-27.7.655 PMC9514759

[46] Siedlecki A, Irish W, Brennan DC. Delayed graft function in the kidney transplant. Am J Transplant 2011; 11:2279–96. 21929642 10.1111/j.1600-6143.2011.03754.x PMC3280444

[47] Prohn M, Viberg A, Zhang D, et al Population pharmacokinetics of letermovir following oral and intravenous administration in healthy participants and allogeneic hematopoietic cell transplantation recipients. CPT Pharmacometrics Syst Pharmacol 2021; 10:255–67. 33440077 10.1002/psp4.12593 PMC7965833

[48] Zhang H, Nguyen MH, Clancy CJ, et al Pharmacokinetics of posaconazole suspension in lung transplant patients with and without cystic fibrosis. Antimicrob Agents Chemother 2016; 60:3558–62. 27021324 10.1128/AAC.00424-16 PMC4879430

[49] Lindsay J, Krantz EM, Morris J, et al Voriconazole in hematopoietic stem cell transplantation and cellular therapies: real-world usage and therapeutic level attainment at a major transplantation center. Transplant Cell Ther 2022; 28:511.e1–e10. 10.1016/j.jtct.2022.05.030 35623614

[50] Selby PR, Heffernan AJ, Yeung D, et al Population pharmacokinetics of posaconazole in allogeneic haematopoietic stem cell transplant patients. J Antimicrob Chemother 2024; 79:567–77. 38217845 10.1093/jac/dkae006

[51] Vanstraelen K, Prattes J, Maertens J, et al Posaconazole plasma exposure correlated to intestinal mucositis in allogeneic stem cell transplant patients. Eur J Clin Pharmacol 2016; 72:953–63. 27066958 10.1007/s00228-016-2057-6

[52] Li M, Zhao Y, Humar A, et al Pharmacokinetics of drugs in adult living donor liver transplant patients: regulatory factors and observations based on studies in animals and humans. Expert Opin Drug Metab Toxicol 2016; 12:231–43. 26809188 10.1517/17425255.2016.1139575

[53] Venkataramanan R, Habucky K, Burckart GJ, et al Clinical pharmacokinetics in organ transplant patients. Clin Pharmacokinet 1989; 16:134–61. 2656047 10.2165/00003088-198916030-00002

[54] Buijk SE . Perioperative pharmacokinetics of cefotaxime in serum and bile during continuous and intermittent infusion in liver transplant patients. J Antimicrob Chemother 2004; 54:199–205. 15175266 10.1093/jac/dkh268

[55] Lin X-B, Li Z, Yan M, et al Population pharmacokinetics of voriconazole and CYP2C19 polymorphisms for optimizing dosing regimens in renal transplant recipients. Br J Clin Pharmacol 2018; 84:1587–97. 29607533 10.1111/bcp.13595 PMC6005582

[56] Cojutti P, Pai MP, Pea F. Population pharmacokinetics and dosing considerations for the use of linezolid in overweight and obese adult patients. Clin Pharmacokinet 2018; 57:989–1000. 29080937 10.1007/s40262-017-0606-5

[57] Suetsugu K, Muraki S, Fukumoto J, et al Effects of letermovir and/or methylprednisolone coadministration on voriconazole pharmacokinetics in hematopoietic stem cell transplantation: a population pharmacokinetic study. Drugs R D 2021; 21:419–29. 34655050 10.1007/s40268-021-00365-0 PMC8602551

[58] Tasnif Y, Morado J, Hebert MF. Pregnancy-related pharmacokinetic changes. Clin Pharmacol Ther 2016; 100:53–62. 27082931 10.1002/cpt.382

[59] Dallmann A, Ince I, Meyer M, et al Gestation-specific changes in the anatomy and physiology of healthy pregnant women: an extended repository of model parameters for physiologically based pharmacokinetic modeling in pregnancy. Clin Pharmacokinet 2017; 56:1303–30. 28401479 10.1007/s40262-017-0539-z

[60] Andrew MA, Easterling TR, Carr DB, et al Amoxicillin pharmacokinetics in pregnant women: modeling and simulations of dosage strategies. Clin Pharmacol Ther 2007; 81:547–56. 17329990 10.1038/sj.clpt.6100126

[61] Hesse MR, Prins JR, Hooge MNL, et al Pharmacokinetics and target attainment of antimicrobial drugs throughout pregnancy: part I—penicillins. Clin Pharmacokinet 2023; 62:221–47. 36662480 10.1007/s40262-023-01211-z PMC9998600

[62] Costantine MM . Physiologic and pharmacokinetic changes in pregnancy. Front Pharmacol 2014; 5:65. 24772083 10.3389/fphar.2014.00065 PMC3982119

[63] Pariente G, Leibson T, Carls A, et al Pregnancy-associated changes in pharmacokinetics: a systematic review. PLoS Med 2016; 13:e1002160. 27802281 10.1371/journal.pmed.1002160 PMC5089741

[64] Heikkilä A, Pyykko K, Erkkola R, et al The pharmacokinetics of mecillinam and pivmecillinam in pregnant and non-pregnant women. Br J Clin Pharmacol 1992; 33:629–33. 1389936 10.1111/j.1365-2125.1992.tb04092.x PMC1381355

[65] Anderson GD . Pregnancy-induced changes in pharmacokinetics: a mechanistic-based approach. Clin Pharmacokinet 2005; 44:989–1008. 16176115 10.2165/00003088-200544100-00001

[66] Groen F, Prins JR, Hooge MNL, et al The pharmacokinetics and target attainment of antimicrobial drugs throughout pregnancy: part III non-penicillin and non-cephalosporin drugs. Clin Pharmacokinet 2023; 62:399–434. 36940039 10.1007/s40262-023-01226-6 PMC10042773

[67] Aoyama H, Izawa Y, Nishizaki A, et al Studies on systemic absorption of tobramycin in polyethylene glycol ointment applied to wounds of burn patients. Burns Incl Therm Inj 1986; 12:153–60. 3708410 10.1016/0305-4179(86)90152-x

[68] Russavage JM, Slater H, Goldfarb IW, et al Penetration of antibiotics through omiderm in vitro and on split-thickness skin grafts in burn patients. Ann Plast Surg 1991; 27:559–61. 1793242 10.1097/00000637-199112000-00008

[69] Price CI, Horton JW, Baxter CR. Liposome delivery of aminoglycosides in burn wounds. Surg Gynecol Obstet 1992; 174:414–8. 1570621

[70] Udy AA, Roberts JA, Lipman J, et al The effects of major burn related pathophysiological changes on the pharmacokinetics and pharmacodynamics of drug use: an appraisal utilizing antibiotics. Adv Drug Deliv Rev 2018; 123:65–74. 28964882 10.1016/j.addr.2017.09.019

[71] Pruskowski KA . Pharmacokinetics and pharmacodynamics of antimicrobial agents in burn patients. Surg Infect (Larchmt) 2021; 22:77–82. 33164665 10.1089/sur.2020.375

[72] Selig DJ, Akers KS, Chung KK, et al Comparison of piperacillin and tazobactam pharmacokinetics in critically ill patients with trauma or with burn. Antibiotics 2022; 11:618. 35625262 10.3390/antibiotics11050618 PMC9138153

[73] Weinbren MJ . Pharmacokinetics of antibiotics in burn patients. J Antimicrob Chemother 1999; 44:319–27. 10511398 10.1093/jac/44.3.319

[74] Jeschke MG, Gauglitz GG, Kulp GA, et al Long-term persistence of the pathophysiologic response to severe burn injury. PLoS One 2011; 6:e21245. 21789167 10.1371/journal.pone.0021245 PMC3138751

[75] Olofsson SK, Marcusson LL, Stromback A, et al Dose-related selection of fluoroquinolone-resistant *Escherichia coli*. J Antimicrob Chemother 2007; 60:795–801. 17635875 10.1093/jac/dkm265

[76] Thomas JK, Forrest A, Bhavnani SM, et al Pharmacodynamic evaluation of factors associated with the development of bacterial resistance in acutely ill patients during therapy. Antimicrob Agents Chemother 1998; 42:521–7. 9517926 10.1128/aac.42.3.521 PMC105492

[77] Jones TW, Jun AH, Michal JL, et al High-dose daptomycin and clinical applications. Ann Pharmacother 2021; 55:1363–78. 33535792 10.1177/1060028021991943 PMC8573721

[78] Chen IH, Nicolau DP. Augmented renal clearance and how to augment antibiotic dosing. Antibiotics 2020; 9:393. 32659898 10.3390/antibiotics9070393 PMC7399877

[79] Mabilat C, Gros MF, Nicolau D, et al Diagnostic and medical needs for therapeutic drug monitoring of antibiotics. Eur J Clin Microbiol Infect Dis 2020; 39:791–7. 31828686 10.1007/s10096-019-03769-8 PMC7182631

[80] Katsube T, Echols R, Wajima T. Pharmacokinetic and pharmacodynamic profiles of cefiderocol, a novel siderophore cephalosporin. Clin Infect Dis 2019; 69:S552–8. 31724042 10.1093/cid/ciz828 PMC6853762

[81] Jones GR . Estimating renal function for drug dosing decisions. Clin Biochem Rev 2011; 32:81–8. 21611081 PMC3100285

[82] Roberts DM, Sevastos J, Carland JE, et al Clinical pharmacokinetics in kidney disease: application to rational design of dosing regimens. Clin J Am Soc Nephrol 2018; 13:1254–63. 30042221 10.2215/CJN.05150418 PMC6086693

[83] Crass RL, Rodvold KA, Mueller BA, et al Renal dosing of antibiotics: are we jumping the gun? Clin Infect Dis 2019; 68:1596–602. 30219824 10.1093/cid/ciy790

[84] Ostermann M, Zarbock A, Goldstein S, et al Recommendations on acute kidney injury biomarkers from the acute disease quality initiative consensus conference: a consensus statement. JAMA Netw Open 2020; 3:e2019209. 33021646 10.1001/jamanetworkopen.2020.19209

[85] Heffernan AJ, Mohd Sazlly Lim S, Lipman J, et al A personalised approach to antibiotic pharmacokinetics and pharmacodynamics in critically ill patients. Anaesth Crit Care Pain Med 2021; 40:100970. 34728411 10.1016/j.accpm.2021.100970

[86] Fiore M, Peluso L, Taccone FS, et al The impact of continuous renal replacement therapy on antibiotic pharmacokinetics in critically ill patients. Expert Opin Drug Metab Toxicol 2021; 17:543–54. 33733979 10.1080/17425255.2021.1902985

[87] Fleming GM . Renal replacement therapy review: past, present and future. Organogenesis 2011; 7:2–12. 21289478 10.4161/org.7.1.13997 PMC3082028

[88] Roberts JA, Bellomo R, Cotta MO, et al Machines that help machines to help patients: optimising antimicrobial dosing in patients receiving extracorporeal membrane oxygenation and renal replacement therapy using dosing software. Intensive Care Med 2022; 48:1338–51. 35997793 10.1007/s00134-022-06847-2 PMC9467945

[89] Pistolesi V, Morabito S, Di Mario F, et al A guide to understanding antimicrobial drug dosing in critically ill patients on renal replacement therapy. Antimicrob Agents Chemother 2019; 63:e00583-19. 31109983 10.1128/AAC.00583-19 PMC6658763

[90] Fouad A, Kobic E, Nicolasora NP, et al Validation of cefiderocol package insert dosing recommendation for patients receiving continuous renal replacement therapy: a prospective multicenter pharmacokinetic study. Open Forum Infect Dis 2024; 11:ofae451. 10.1093/ofid/ofae451 PMC11492798 39435320

[91] Wenzler E, Butler D, Tan X, et al Pharmacokinetics, pharmacodynamics, and dose optimization of cefiderocol during continuous renal replacement therapy. Clin Pharmacokinet 2022; 61:539–52. 34792787 10.1007/s40262-021-01086-y PMC9167810

[92] Kois AK, Gluck JA, Nicolau DP, et al Pharmacokinetics and time above the MIC exposure of cefepime in critically ill patients receiving extracorporeal membrane oxygenation (ECMO). Int J Antimicrob Agents 2022; 60:106603. 35577257 10.1016/j.ijantimicag.2022.106603

[93] Hahn J, Min KL, Kang S, et al Population pharmacokinetics and dosing optimization of piperacillin-tazobactam in critically ill patients on extracorporeal membrane oxygenation and the influence of concomitant renal replacement therapy. Microbiol Spectr 2021; 9:e0063321. 34937189 10.1128/Spectrum.00633-21 PMC8694146

[94] Bakdach D, Elajez R, Bakdach AR, et al Pharmacokinetics, pharmacodynamics, and dosing considerations of novel β-lactams and β-lactam/β-lactamase inhibitors in critically ill adult patients: focus on obesity, augmented renal clearance, renal replacement therapies, and extracorporeal membrane oxygenation. J Clin Med 2022; 11:6898. 36498473 10.3390/jcm11236898 PMC9738279

[95] Richter DC, Frey O, Röhr A, et al Therapeutic drug monitoring-guided continuous infusion of piperacillin/tazobactam significantly improves pharmacokinetic target attainment in critically ill patients: a retrospective analysis of four years of clinical experience. Infection 2019; 47:1001–11. 31473974 10.1007/s15010-019-01352-z

[96] Gatti M, Cojutti PG, Pascale R, et al Assessment of a PK/PD target of continuous infusion beta-lactams useful for preventing microbiological failure and/or resistance development in critically ill patients affected by documented gram-negative infections. Antibiotics 2021; 10:1311. 34827249 10.3390/antibiotics10111311 PMC8615220

[97] Guilhaumou R, Chevrier C, Setti JL, et al β-Lactam pharmacokinetic/pharmacodynamic target attainment in intensive care unit patients: a prospective, observational, cohort study. Antibiotics 2023; 12:1289. 37627709 10.3390/antibiotics12081289 PMC10451857

[98] Heil EL, Nicolau DP, Farkas A, et al Pharmacodynamic target attainment for cefepime, meropenem, and piperacillin-tazobactam using a pharmacokinetic/pharmacodynamic-based dosing calculator in critically ill patients. Antimicrob Agents Chemother 2018; 62:e01008-18. 29967022 10.1128/AAC.01008-18 PMC6125501

[99] Stierman B, Afful J, Carroll MD, et al National Health and Nutrition Examination Survey 2017–March 2020 prepandemic data files—development of files and prevalence estimates for selected health outcomes. Natl Health Stat Report 2021:1–20. 10.15620/cdc:106273 PMC11513744 39380201

[100] Centers for Disease Control and Prevention (CDC) . Defining adult overweight and obesity. Atlanta, GA: CDC, 2022.

[101] Shank BZ . Demystifying drug dosing in obese patients. Bethesda, MD: American Society of Health-System Pharmacists, 2015.

[102] Cockshott WP, Thompson GT, Howlett LJ, et al Intramuscular or intralipomatous injections? N Engl J Med 1982; 307:356–8. 7088101 10.1056/NEJM198208053070607

[103] Haselden M, Leach M, Bohm N. Daptomycin dosing strategies in patients receiving thrice-weekly intermittent hemodialysis. Ann Pharmacother 2013; 47:1342–7. 24259698 10.1177/1060028013503110

[104] Boonpeng A, Jaruratanasirikul S, Jullangkoon M, et al Population pharmacokinetics/pharmacodynamics and clinical outcomes of meropenem in critically ill patients. Antimicrob Agents Chemother 2022; 66:e0084522. 36226944 10.1128/aac.00845-22 PMC9664862

[105] Crandon JL, Ariano RE, Zelenitsky SA, et al Optimization of meropenem dosage in the critically ill population based on renal function. Intensive Care Med 2011; 37:632–8. 21136037 10.1007/s00134-010-2105-0

[106] Turner RB, Cumpston A, Sweet M, et al Prospective, controlled study of acyclovir pharmacokinetics in obese patients. Antimicrob Agents Chemother 2016; 60:1830–3. 26824940 10.1128/AAC.02010-15 PMC4775958

[107] Barber KE, Wagner JL, Stover KR. Impact of obesity on acyclovir-induced nephrotoxicity. Open Forum Infect Dis 2019; 6:ofz121. 31024972 10.1093/ofid/ofz121 PMC6475582

[108] Wong A, Pickering AJ, Potoski BA. Dosing practices of intravenous acyclovir for herpes encephalitis in obesity: results of a pharmacist survey. J Pharm Pract 2017; 30:324–8. 27067742 10.1177/0897190016642689

[109] Swank ML, Wing DA, Nicolau DP, et al Increased 3-gram cefazolin dosing for cesarean delivery prophylaxis in obese women. Am J Obstet Gynecol 2015; 213:415.e1–8. 10.1016/j.ajog.2015.05.030 26003059

[110] Grupper M, Kuti JL, Swank ML, et al Population pharmacokinetics of cefazolin in serum and adipose tissue from overweight and obese women undergoing cesarean delivery. J Clin Pharmacol 2017; 57:712–9. 27925657 10.1002/jcph.851

[111] Bratzler DW, Dellinger EP, Olsen KM, et al Clinical practice guidelines for antimicrobial prophylaxis in surgery. Am J Health Syst Pharm 2013; 70:195–283. 23327981 10.2146/ajhp120568

[112] Coates M, Shield A, Peterson GM, et al Prophylactic cefazolin dosing in obesity—a systematic review. Obes Surg 2022; 32:3138–49. 35809198 10.1007/s11695-022-06196-5 PMC9392691

[113] Payne KD, Hall RG. Dosing of antibacterial agents in obese adults: does one size fit all? Expert Rev Anti Infect Ther 2014; 12:829–54. 24809811 10.1586/14787210.2014.912942

[114] Blackman AL, Jarugula P, Nicolau DP, et al Evaluation of linezolid pharmacokinetics in critically ill obese patients with severe skin and soft tissue infections. Antimicrob Agents Chemother 2021; 65:e01619-20. 33257446 10.1128/AAC.01619-20 PMC7848975

[115] Simon P, Busse D, Petroff D, et al Linezolid concentrations in plasma and subcutaneous tissue are reduced in obese patients, resulting in a higher risk of underdosing in critically ill patients: a controlled clinical pharmacokinetic study. J Clin Med 2020; 9:1067. 32283731 10.3390/jcm9041067 PMC7230366

[116] Corcione S, Pagani N, Baietto L, et al Pharmacokinetics of high dosage of linezolid in two morbidly obese patients. J Antimicrob Chemother 2015; 70:2417–8. 25957383 10.1093/jac/dkv126

[117] Janson B, Thursky K. Dosing of antibiotics in obesity. Curr Opin Infect Dis 2012; 25:634–49. 23041773 10.1097/QCO.0b013e328359a4c1

[118] Meng L, Mui E, Holubar MK, et al Comprehensive guidance for antibiotic dosing in obese adults. Pharmacotherapy 2017; 37:1415–31. 28869666 10.1002/phar.2023

[119] Xie F, Mantzarlis K, Malliotakis P, et al Pharmacokinetic evaluation of linezolid administered intravenously in obese patients with pneumonia. J Antimicrob Chemother 2019; 74:667–74. 30535122 10.1093/jac/dky500

[120] Payne KD, Hall RG. Dosing of antifungal agents in obese people. Expert Rev Anti Infect Ther 2016; 14:257–67. 26641135 10.1586/14787210.2016.1128822

[121] Richards PG, Dang KM, Kauffman CA, et al Therapeutic drug monitoring and use of an adjusted body weight strategy for high-dose voriconazole therapy. J Antimicrob Chemother 2017; 72:1178–83. 28108679 10.1093/jac/dkw550

[122] Koselke E, Kraft S, Smith J, et al Evaluation of the effect of obesity on voriconazole serum concentrations. J Antimicrob Chemother 2012; 67:2957–62. 22915462 10.1093/jac/dks312

[123] Diller E, Krekel T, Spec A, et al Evaluation of total body weight versus adjusted body weight voriconazole dosing in obese patients. Antimicrob Agents Chemother 2021; 65:e0246020. 33875427 10.1128/AAC.02460-20 PMC8218689

[124] Davies-Vorbrodt S, Ito J I, Tegtmeier BR, et al Voriconazole serum concentrations in obese and overweight immunocompromised patients: a retrospective review. Pharmacotherapy 2013; 33:22–30. 23307541 10.1002/phar.1156

[125] Barsky EE, Pereira LM, Sullivan KJ, et al Ceftaroline pharmacokinetics and pharmacodynamics in patients with cystic fibrosis. J Cyst Fibros 2018; 17:e25–31. 29103924 10.1016/j.jcf.2017.10.010

[126] Panel on Treatment of HIV During Pregnancy and Prevention of Perinatal Transmission. Safety and Toxicity of Individual Antiretroviral Agents in Pregnancy. ClinicalInfo.HIV.gov. National Institutes of Health. https://clinicalinfo.hiv.gov/en/guidelines/perinatal/safety-toxicity-arv-agents-drug-use-pregnant-full. Accessed 4 March 2026.

[127] Torian SC, Wiktor AJ, Roper SE, et al Burn injury and augmented renal clearance: a case for optimized piperacillin-tazobactam dosing. J Burn Care Res 2023; 44:203–6. 36173707 10.1093/jbcr/irac138 PMC9825348

[128] Cota JM, FakhriRavari A, Rowan MP, et al Intravenous antibiotic and antifungal agent pharmacokinetic-pharmacodynamic dosing in adults with severe burn injury. Clin Ther 2016; 38:2016–31. 27586127 10.1016/j.clinthera.2016.08.001

[129] Varela JE, Cohn SM, Brown M, et al Pharmacokinetics and burn eschar penetration of intravenous ciprofloxacin in patients with major thermal injuries. J Antimicrob Chemother 2000; 45:337–42. 10702553 10.1093/jac/45.3.337

[130] Garrelts JC, Jost G, Kowalsky SF, et al Ciprofloxacin pharmacokinetics in burn patients. Antimicrob Agents Chemother 1996; 40:1153–6. 8723457 10.1128/aac.40.5.1153 PMC163282

[131] Lesne-Hulin A, Bourget P, Ravat F, et al Clinical pharmacokinetics of ciprofloxacin in patients with major burns. Eur J Clin Pharmacol 1999; 55:515–9. 10501821 10.1007/s002280050666

[132] Pinner NA, Tapley NG, Barber KE, et al Effect of obesity on clinical failure of patients treated with β-lactams. Open Forum Infect Dis 2021; 8:ofab212. 34458387 10.1093/ofid/ofab212 PMC8391092

[133] Davies SW, Efird JT, Guidry CA, et al Vancomycin-associated nephrotoxicity: the obesity factor. Surg Infect (Larchmt) 2015; 16:684–93. 26324996 10.1089/sur.2014.198 PMC4663651

[134] Gerber JS, Kronman MP, Ross RK, et al Identifying targets for antimicrobial stewardship in children's hospitals. Infect Control Hosp Epidemiol 2013; 34:1252–8. 24225609 10.1086/673982

[135] Waters VJ, Ratjen FA. Is there a role for antimicrobial stewardship in cystic fibrosis? Ann Am Thorac Soc 2014; 11:1116–9. 25102101 10.1513/AnnalsATS.201401-034OI

[136] El Hassani M, Caissy J-A, Marsot A. Antibiotics in adult cystic fibrosis patients: a review of population pharmacokinetic analyses. Clin Pharmacokinet 2021; 60:447–70. 33447944 10.1007/s40262-020-00970-3

[137] Magréault S, Roy C, Launay M, et al Pharmacokinetic and pharmacodynamic optimization of antibiotic therapy in cystic fibrosis patients: current evidences, gaps in knowledge and future directions. Clin Pharmacokinet 2021; 60:409–45. 33486720 10.1007/s40262-020-00981-0

[138] Akkerman-Nijland AM, Akkerman OW, Grasmeijer F, et al The pharmacokinetics of antibiotics in cystic fibrosis. Expert Opin Drug Metab Toxicol 2021; 17:53–68. 33213220 10.1080/17425255.2021.1836157

[139] Tam RY, van Dorst JM, McKay I, et al Intestinal inflammation and alterations in the gut microbiota in cystic fibrosis: a review of the current evidence, pathophysiology and future directions. J Clin Med 2022; 11:649. 35160099 10.3390/jcm11030649 PMC8836727

[140] Keel RA, Schaeftlein A, Kloft C, et al Pharmacokinetics of intravenous and oral linezolid in adults with cystic fibrosis. Antimicrob Agents Chemother 2011; 55:3393–8. 21518837 10.1128/AAC.01797-10 PMC3122391

[141] Epps QJ, Epps KL, Young DC, et al State of the art in cystic fibrosis pharmacology-optimization of antimicrobials in the treatment of cystic fibrosis pulmonary exacerbations: I. Anti–methicillin-resistant *Staphylococcus aureus* (MRSA) antibiotics. Pediatr Pulmonol 2020; 55:33–57. 31609097 10.1002/ppul.24537

[142] Zobell JT, Young DC, Waters CD, et al Optimization of anti-pseudomonal antibiotics for cystic fibrosis pulmonary exacerbations: VI. Executive summary. Pediatr Pulmonol 2013; 48:525–37. 23359557 10.1002/ppul.22757

[143] Mitchell B, Kormelink L, Kuhn R, et al Retrospective review of vancomycin monitoring via trough only versus two-point estimated area under the curve in pediatric and adult patients with cystic fibrosis. Pediatr Pulmonol 2023; 58:239–45. 36203329 10.1002/ppul.26190

[144] Butterfield JM, Lodise TP, Beegle S, et al Pharmacokinetics and pharmacodynamics of extended-infusion piperacillin/tazobactam in adult patients with cystic fibrosis-related acute pulmonary exacerbations. J Antimicrob Chemother 2014; 69:176–9. 23869050 10.1093/jac/dkt300

[145] Davis SE, Ham J, Hucks J, et al Use of continuous infusion ceftolozane-tazobactam with therapeutic drug monitoring in a patient with cystic fibrosis. Am J Health Syst Pharm 2019; 76:501–4. 31361864 10.1093/ajhp/zxz011

[146] Monogue ML, Pettit RS, Muhlebach M, et al Population pharmacokinetics and safety of ceftolozane-tazobactam in adult cystic fibrosis patients admitted with acute pulmonary exacerbation. Antimicrob Agents Chemother 2016; 60:6578–84. 27550351 10.1128/AAC.01566-16 PMC5075062

[147] Taccetti G, Francalanci M, Pizzamiglio G, et al Cystic fibrosis: recent insights into inhaled antibiotic treatment and future perspectives. Antibiotics (Basel) 2021; 10:338. 33810116 10.3390/antibiotics10030338 PMC8004710

[148] Labiris NR, Dolovich MB. Pulmonary drug delivery. Part I: physiological factors affecting therapeutic effectiveness of aerosolized medications. Br J Clin Pharmacol 2003; 56:588–99. 14616418 10.1046/j.1365-2125.2003.01892.x PMC1884307

[149] Eedara BB, Bastola R, Das SC. Dissolution and absorption of inhaled drug particles in the lungs. Pharmaceutics 2022; 14:2667. 36559160 10.3390/pharmaceutics14122667 PMC9781681

[150] McKinzie CJ, Chen L, Ehlert K, et al Off-label use of intravenous antimicrobials for inhalation in patients with cystic fibrosis. Pediatr Pulmonol 2019; 54:S27–45. 31715085 10.1002/ppul.24511

[151] Cogen JD, Nichols DP, Goss CH, et al Drugs, drugs, drugs: current treatment paradigms in cystic fibrosis airway infections. J Pediatric Infect Dis Soc 2022; 11:S32–9. 36069901 10.1093/jpids/piac061 PMC10233481

[152] Cvetkovich-Muntanola A . The changing landscape of cystic fibrosis clinical trials. Applied Clinical Trials. Published June 1, 2013. https://www.appliedclinicaltrialsonline.com/view/changing-landscape-cystic-fibrosis-clinical-trials. Accessed 4 March 2026.

[153] Stillhart C, Vučićević K, Augustijns P, et al Impact of gastrointestinal physiology on drug absorption in special populations—an UNGAP review. Eur J Pharm Sci 2020; 147:105280. 32109493 10.1016/j.ejps.2020.105280

[154] Labriffe M, Vaidie J, Monchaud C, et al Population pharmacokinetics and Bayesian estimators for intravenous mycophenolate mofetil in haematopoietic stem cell transplant patients. Br J Clin Pharmacol 2020; 86:1550–9. 32073158 10.1111/bcp.14261 PMC7373706

[155] Han K, Capitano B, Bies R, et al Bioavailability and population pharmacokinetics of voriconazole in lung transplant recipients. Antimicrob Agents Chemother 2010; 54:4424–31. 20679503 10.1128/AAC.00504-10 PMC2944566

[156] Selby PR, Shakib S, Peake SL, et al A systematic review of the clinical pharmacokinetics, pharmacodynamics and toxicodynamics of ganciclovir/valganciclovir in allogeneic haematopoietic stem cell transplant patients. Clin Pharmacokinet 2021; 60:727–39. 33515202 10.1007/s40262-020-00982-z

[157] Brooks E, Tett SE, Isbel NM, et al Population pharmacokinetic modelling and Bayesian estimation of tacrolimus exposure: is this clinically useful for dosage prediction yet? Clin Pharmacokinet 2016; 55:1295–335. 27138787 10.1007/s40262-016-0396-1

[158] Campagne O, Mager DE, Brazeau D, et al Tacrolimus population pharmacokinetics and multiple CYP3A5 genotypes in black and white renal transplant recipients. J Clin Pharmacol 2018; 58:1184–95. 29775201 10.1002/jcph.1118 PMC6105387

[159] Erbas B . Peri- and postsurgical evaluations of renal transplant. Semin Nucl Med 2017; 47:647–59. 28969763 10.1053/j.semnuclmed.2017.07.002

[160] Rodrigo E, Fernández-Fresnedo G, Ruiz JC, et al Similar impact of slow and delayed graft function on renal allograft outcome and function. Transplant Proc 2005; 37:1431–2. 15866627 10.1016/j.transproceed.2005.02.052

[161] Johnston O, O’Kelly P, Spencer S, et al Reduced graft function (with or without dialysis) vs immediate graft function—a comparison of long-term renal allograft survival. Nephrol Dialysis Transplant 2006; 21:2270–4. 10.1093/ndt/gfl103 16720598

[162] Baker RJ, Mark PB, Patel RK, et al Renal association clinical practice guideline in post-operative care in the kidney transplant recipient. BMC Nephrol 2017; 18:174. 28571571 10.1186/s12882-017-0553-2 PMC5455080

[163] Avram MJ . Pharmacokinetic studies in pregnancy. Semin Perinatol 2020; 44:151227. 32093881 10.1016/j.semperi.2020.151227 PMC7323629

[164] Moreira FL, Benzi JRL, Pinto L, et al Optimizing therapeutic drug monitoring in pregnant women: a critical literature review. Ther Drug Monit 2023; 45:159–72. 36127797 10.1097/FTD.0000000000001039

[165] Cox E, Nambiar S, Baden L. Needed: antimicrobial development. N Engl J Med 2019; 380:783–5. 30786194 10.1056/NEJMe1901525

[166] Bassetti M, Echols R, Matsunaga Y, et al Efficacy and safety of cefiderocol or best available therapy for the treatment of serious infections caused by carbapenem-resistant gram-negative bacteria (CREDIBLE-CR): a randomised, open-label, multicentre, pathogen-focused, descriptive, phase 3 trial. Lancet Infect Dis 2021; 21:226–40. 33058795 10.1016/S1473-3099(20)30796-9

[167] Wunderink RG, Giamarellos-Bourboulis EJ, Rahav G, et al Effect and safety of meropenem-vaborbactam versus best-available therapy in patients with carbapenem-resistant Enterobacteriaceae infections: the TANGO II randomized clinical trial. Infect Dis Ther 2018; 7:439–55. 30270406 10.1007/s40121-018-0214-1 PMC6249182

[168] Choradia N, Karzai F, Nipp R, et al Increasing diversity in clinical trials: demographic trends at the National Cancer Institute, 2005–2020. J Natl Cancer Inst 2024; 116:1063–71. 38374401 10.1093/jnci/djae018 PMC11223850

[169] US Food and Drug Administration (FDA) . Exposure–response relationships—study design, data analysis, and regulatory applications. Silver Spring, MD: FDA, 2003.

[170] Center for Drug Evaluation and Research . Qualified infectious disease product designation questions and answers. Silver Spring, MD: US Food and Drug Administration, 2021.

[171] Stets R, Popescu M, Gonong JR, et al Omadacycline for community-acquired bacterial pneumonia. N Engl J Med 2019; 380:517–27. 30726692 10.1056/NEJMoa1800201

[172] Bax HI, de Vogel CP, Mouton JW, et al Omadacycline as a promising new agent for the treatment of infections with *Mycobacterium abscessus*. J Antimicrob Chemother 2019; 74:2930–3. 31236595 10.1093/jac/dkz267 PMC7183808

[173] Center for Drug Evaluation and Research . Office of Infectious Diseases Research Activities. Silver Spring, MD: US Food and Drug Administration, 2023.

[174] Center for Drug Evaluation and Research . Pharmacokinetics in patients with impaired renal function—study design, data analysis, and impact on dosing and labeling. Silver Spring, MD: US Food and Drug Administration, 2020.

[175] Bohn B, et al . SIDP position on incentives to support antimicrobial drug development. Warrenville, IL: Society of Infectious Diseases Pharmacists, 2024.

[176] Cystic Fibrosis Foundation. Introduction to the Therapeutics Development Network. Cystic Fibrosis Foundation website. https://www.cff.org/researchers/introduction-therapeutics-development-network. Accessed 12 March 2026.

[177] Crass RL, Cojutti PG, Pai MP, et al, Reappraisal of linezolid dosing in renal impairment to improve safety. Antimicrob Agents Chemother 2019; 63:e00605-19. 31109977 10.1128/AAC.00605-19 PMC6658752

[178] Shorr AF, Bruno CJ, Zhang Z, et al Ceftolozane/tazobactam probability of target attainment and outcomes in participants with augmented renal clearance from the randomized phase 3 ASPECT-NP trial. Crit Care 2021; 25:354. 34600585 10.1186/s13054-021-03773-5 PMC8487337

[179] Bitterman R, Koppel F, Mussini C, et al Piperacillin-tazobactam versus meropenem for treatment of bloodstream infections caused by third-generation cephalosporin-resistant Enterobacteriaceae: a study protocol for a non-inferiority open-label randomised controlled trial (PeterPen). BMJ Open 2021; 11:e040210. 10.1136/bmjopen-2020-040210 PMC7871690 33558347

[180] Churchwell MD . Use of an in vitro model of renal replacement therapy systems to estimate extracorporeal drug removal. J Clin Pharmacol 2012; 52:35S–44S. 22232751 10.1177/0091270011415979

[181] Onita T, Ishihara N, Yano T. PK/PD-guided strategies for appropriate antibiotic use in the era of antimicrobial resistance. Antibiotics 2025; 14:92. 39858377 10.3390/antibiotics14010092 PMC11759776

[182] Koenig C, McGrath CP, Roenfanz HF, et al Clinical validation of antimicrobial dosing regimens for continuous renal replacement therapy based on an ex vivo dosing algorithm. J Antimicrob Chemother 2025; 80:2269–79. 40576018 10.1093/jac/dkaf199

[183] McGovern PC, Wible M, El-Tahtawy A, et al All-cause mortality imbalance in the tigecycline phase 3 and 4 clinical trials. Int J Antimicrob Agents 2013; 41:463–7. 23537581 10.1016/j.ijantimicag.2013.01.020

[184] Abdul-Aziz MH, Alffenaar J-WC, Bassetti M, et al Antimicrobial therapeutic drug monitoring in critically ill adult patients: a position paper. Intensive Care Med 2020; 46:1127–53. 32383061 10.1007/s00134-020-06050-1 PMC7223855

[185] Arends A, Pettit R. Safety of extended interval tobramycin in cystic fibrosis patients less than 6 years old. J Pediatr Pharmacol Ther 2018; 23:152–8. 29720918 10.5863/1551-6776-23.2.152 PMC5916444

[186] Hagel S, Bach F, Brenner T, et al Effect of therapeutic drug monitoring-based dose optimization of piperacillin/tazobactam on sepsis-related organ dysfunction in patients with sepsis: a randomized controlled trial. Intensive Care Med 2022; 48:311–21. 35106617 10.1007/s00134-021-06609-6 PMC8866359

[187] Abdulla A, Ewoldt TMJ, Hunfeld NGM, et al The effect of therapeutic drug monitoring of beta-lactam and fluoroquinolones on clinical outcome in critically ill patients: the DOLPHIN trial protocol of a multi-centre randomised controlled trial. BMC Infect Dis 2020; 20:57. 31952493 10.1186/s12879-020-4781-x PMC6969462

[188] Al-Shaer MH, Rubido E, Cherabuddi K, et al Early therapeutic monitoring of β-lactams and associated therapy outcomes in critically ill patients. J Antimicrob Chemother 2020; 75:3644–51. 32910809 10.1093/jac/dkaa359

[189] Flume PA, Mogayzel PJ, Robinson KA, et al Cystic fibrosis pulmonary guidelines. Am J Respir Crit Care Med 2009; 180:802–8. 19729669 10.1164/rccm.200812-1845PP

[190] Tamma PD, Heil EL, Justo JA, et al Infectious Diseases Society of America 2024 guidance on the treatment of antimicrobial-resistant gram-negative infections [manuscript published online ahead of print 7 August 2024]. Clin Infect Dis 2024. doi:10.1093/cid/ciae403 39108079

[191] Kalil AC, Metersky ML, Klompas M, et al Management of adults with hospital-acquired and ventilator-associated pneumonia: 2016 clinical practice guidelines by the Infectious Diseases Society of America and the American Thoracic Society. Clin Infect Dis 2016; 63:e61–111. 27418577 10.1093/cid/ciw353 PMC4981759

[192] Hoff BM, Maker JH, Dager WE, et al Antibiotic dosing for critically ill adult patients receiving intermittent hemodialysis, prolonged intermittent renal replacement therapy, and continuous renal replacement therapy: an update. Ann Pharmacother 2020; 54:43–55. 31342772 10.1177/1060028019865873

[193] McGrath C, Koenig C, Roenfanz HF, et al An ex vivo model to determine transmembrane clearance of antimicrobials during continuous renal replacement therapy. J Antimicrob Chemother 2025; 80:2109–16. 40444723 10.1093/jac/dkaf177

[194] Guilhaumou R, Benaboud S, Bennis Y, et al Optimization of the treatment with beta-lactam antibiotics in critically ill patients—guidelines from the French Society of Pharmacology and Therapeutics (Société Française de Pharmacologie et Thérapeutique-SFPT) and the French Society of Anaesthesia and Intensive Care Medicine (Société Française d’Anesthésie et Réanimation-SFAR). Crit Care 2019; 23:104. 30925922 10.1186/s13054-019-2378-9 PMC6441232

[195] Duceppe M-A, Kanji S, Do AT, et al Pharmacokinetics of commonly used antimicrobials in critically ill adults during extracorporeal membrane oxygenation: a systematic review. Drugs 2021; 81:1307–29. 34224115 10.1007/s40265-021-01557-3

[196] Dyer CJ, De Waele JJ, Roberts JA. Antibiotic dose optimisation in the critically ill: targets, evidence and future strategies. Curr Opin Crit Care 2024; 30:439–47. doi:10.1097/MCC.0000000000001187 39150038

[197] Kim M, Mahmood M, Estes LL, et al A narrative review on antimicrobial dosing in adult critically ill patients on extracorporeal membrane oxygenation. Crit Care 2024; 28:326. 39367501 10.1186/s13054-024-05101-z PMC11453026

[198] Sanford Guide. Sanford Guide. Antimicrobial Therapy, Inc. https://www.sanfordguide.com/. Accessed 4 March 2026.

[199] Märtson AG, Barber KE, Crass RL, et al The pharmacokinetics of antibiotics in patients with obesity: a systematic review and consensus guidelines for dose adjustments. Lancet Infect Dis 2025; 25:e504–15. 40383125 10.1016/S1473-3099(25)00155-0

[200] Rolsma SL, Sokolow A, Patel PC, et al Population pharmacokinetic modeling of cefepime, meropenem, and piperacillin-tazobactam in patients with cystic fibrosis. J Infect Dis 2025; 231:e364–74. 39344185 10.1093/infdis/jiae451 PMC11841632

[201] Pinheiro EA, Stika CS. Drugs in pregnancy: pharmacologic and physiologic changes that affect clinical care. Semin Perinatol 2020; 44:151221. 32115202 10.1016/j.semperi.2020.151221 PMC8195457

[202] Nguyen J, Madonia V, Bland CM, et al A review of antibiotic safety in pregnancy—2025 update. Pharmacother J Hum Pharmacol Drug Ther 2025; 45:227–37. 10.1002/phar.70010 PMC11998890 40105039

[203] Blanchet B, Jullien V, Vinsonneau C, et al Influence of burns on pharmacokinetics and pharmacodynamics of drugs used in the care of burn patients. Clin Pharmacokinet 2008; 47:635–54. 18783295 10.2165/00003088-200847100-00002

[204] Auger C, Samadi O, Jeschke MG. The biochemical alterations underlying post-burn hypermetabolism. Biochim Biophys Acta Mol Basis Dis 2017; 1863:2633–44. 28219767 10.1016/j.bbadis.2017.02.019 PMC5563481

